# Differential DNA Methylation Regions in Cytokine and Transcription Factor Genomic Loci Associate with Childhood Physical Aggression

**DOI:** 10.1371/journal.pone.0071691

**Published:** 2013-08-19

**Authors:** Nadine Provençal, Matthew J. Suderman, Doretta Caramaschi, Dongsha Wang, Michael Hallett, Frank Vitaro, Richard E. Tremblay, Moshe Szyf

**Affiliations:** 1 Department of Pharmacology and Therapeutics, McGill University, Montreal, Quebec, Canada; 2 Research Unit on Children’s Psycho-Social Maladjustment and Ste-Justine Hospital Research Center, University of Montreal, Montreal, Quebec, Canada; 3 Sackler Program for Epigenetics and Psychobiology, McGill University, Montreal, Quebec, Canada; 4 Department of Psychology and Pediatrics, University of Montreal, Montreal, Quebec, Canada; 5 School of Public Health and Population Science, University College Dublin, Dublin, Ireland; 6 McGill Centre for Bioinformatics, McGill University, Montreal, Quebec, Canada; 7 School of Psycho-Education, University of Montreal, Montreal, Quebec, Canada; The Nathan Kline Institute, United States of America

## Abstract

**Background:**

Animal and human studies suggest that inflammation is associated with behavioral disorders including aggression. We have recently shown that physical aggression of boys during childhood is strongly associated with reduced plasma levels of cytokines IL-1α, IL-4, IL-6, IL-8 and IL-10, later in early adulthood. This study tests the hypothesis that there is an association between differential DNA methylation regions in cytokine genes in T cells and monocytes DNA in adult subjects and a trajectory of physical aggression from childhood to adolescence.

**Methodology/Principal Findings:**

We compared the methylation profiles of the entire genomic loci encompassing the IL-1α, IL-6, IL-4, IL-10 and IL-8 and three of their regulatory transcription factors (TF) NFkB1, NFAT5 and STAT6 genes in adult males on a chronic physical aggression trajectory (CPA) and males with the same background who followed a normal physical aggression trajectory (control group) from childhood to adolescence. We used the method of methylated DNA immunoprecipitation with comprehensive cytokine gene loci and TF loci microarray hybridization, statistical analysis and false discovery rate correction. We found differentially methylated regions to associate with CPA in both the cytokine loci as well as in their transcription factors loci analyzed. Some of these differentially methylated regions were located in known regulatory regions whereas others, to our knowledge, were previously unknown as regulatory areas. However, using the ENCODE database, we were able to identify key regulatory elements in many of these regions that indicate that they might be involved in the regulation of cytokine expression.

**Conclusions:**

We provide here the first evidence for an association between differential DNA methylation in cytokines and their regulators in T cells and monocytes and male physical aggression.

## Introduction

Physical violence is an important health problem, especially among males [1]. The development of physical aggression has been studied with large population based longitudinal studies from birth to adulthood. Results show that children start using physical aggressions by the end of the first year after birth, increase their frequency from 2 to 4 years of age [2]–[5], and then reduce the frequency from school entry to adulthood [6]. However, a minority of children (4–7%), mainly males, maintain a high frequency of physical aggression from childhood to adolescence [5]–[7]. These children tend to be impulsive, hyperactive, oppositional and rejected by their peers, they also tend to fail in school and have serious social adjustment problems during adulthood [8]–[12]. There is good evidence that the parents of children on a high trajectory of physical aggression exhibit similar behavioral problems creating early childhood family environments which do not support learning to regulate physically aggressive reactions [5], [7], [13]–[16].

A growing body of research suggests that inflammatory cytokines might have systemic effects in addition to their role in the immune response. Recent studies have shown that changes in cytokine expression levels are associated with various behavioral disorders such as anxiety, depression, suicide, childhood mood disorder and post-traumatic stress disorder (PTSD) [17]–[28]. In normal men, assessments of hostility, physical aggression, and verbal aggression were positively associated with lipopolysaccharide stimulated TNF-α expression in blood monocytes [29]. Moderate to severe maltreatment during childhood was also observed to be positively correlated with overall change in stress-induced IL-6 concentrations [30]. Other studies examined the association between cytokines and aggression in animals. Gene knockout depletion of IL-6 (−/−) in mice resulted in increased aggression compared to control mice and over-expression of IL-6 in the brain of normal mice increases affiliative behavior [31]. We have recently shown that consistent with these data in mice, physical aggression of boys during childhood is associated with reduced plasma levels of cytokines later in early adulthood [32]. Compared to the control group, men on a chronic physical aggression trajectory from childhood to adolescence had consistently lower plasma levels of five cytokines: pro-inflammatory interleukins IL-1α and IL-6, anti-inflammatory interleukin IL-4 and IL-10, and chemokine IL-8. However, the mechanisms that differentially regulate cytokine expression in white blood cells in chronically aggressive humans are unknown.

DNA methylation is involved in programming cell type specific gene expression during development [33]. Consistent with this developmental role of DNA methylation, it is involved in naive CD4+ T cells differentiation into Th1 and Th2 cells [34], [35]. The Th2 cytokine locus (IL-4-IL-13-Rad50-IL-5 locus) expressed in Th2 and the IFNγ locus expressed in Th1, undergo chromatin remodeling and DNA demethylation during differentiation ([35] and [36] for review). DNA methylation regulates cytokine gene expression (IL-1α [37], IL-6 [38], IL-8 [39], IL-10 [40] and IL-4 [36]) as well as the expression of the transcription factors (TF) that regulate cytokine expression (NFAT5 [41], STAT6 [42] and STAT1 [43]). TFs are also involved in epigenetic reprogramming of their cytokine targets. For example, STATs are required for maintenance of histone acetylation states in the IL-4 and IFNγ locus [44] and establishment of histone acetylation and DNA demethylation in IL-4 locus requires the presence of STAT6 [44], [45].

Evidence is emerging that in addition to its role in regulating gene expression during differentiation, the DNA methylation pattern is responsive to external environmental exposures including the social environment [46] in animals [47]–[54] and in humans [55]–[59]. Importantly, DNA methylation alterations associated with social exposures are not restricted to the brain and are detected in whole blood cell (WBC) DNA [27], [55], [57], [58], [60]–[67]. It is therefore plausible that alteration in DNA methylation of cytokine regulatory regions and the transcription factors that regulate them occur in response to social signals and play a role in human behavior, including aggression.

Previous studies from our group and others that have examined associations of genome wide DNA methylation profiles with behavioral exposures have pointed to immune pathways, particularly cytokines. We have shown that maternal deprivation in rhesus macaques is associated with changes in DNA methylation in promoters regulating genes in the immune response pathways [68]. Specifically, transcription factors, cytokines and their receptors, such as NFkB2, IL-16 and IFNγR2 gene promoters, were identified to be differentially methylated in T cells. In humans, we have described association between early life socioeconomic position (SEP) and adult WBC DNA methylation signatures in chemokines (CCL3, CXCL9, CCR1, CCR10, CCR3, CCR6), interleukins (IL-2, IL-4, IL-8, IL1RN), cytokine receptors (IL10RB, IL6RB) and transcription factors regulating cytokine gene expression (NFATC1, NF-IL3, IkBβ) [60]. DNA methylation alterations in white blood cell DNA that were associated with PTSD, included genes involved in inflammation, such as CLEC9A, ANXA2, TLR8 [27]. Lastly, in subjects with a lifetime history of depression, IL-6 and CRP serum levels were found to be elevated, where IL-6 methylation in WBC DNA showed an inverse correlation with circulating IL-6 and CRP [69].

We have previously reported lower plasma cytokines levels in aggressive subjects [32]. We therefore compared the methylation profiles of IL-1α, IL-6, IL-4, IL-10 and IL-8 gene loci and three of their transcription factors NFkB1, NFAT5 and STAT6 gene loci in adult males on a chronic physical aggression trajectory (CPA) between 6 and 15 years of age and males with the same background who followed a normal physical aggression trajectory (control group). Although the promoter area and the immediate 5′ regulatory regions play an important role in regulation of gene expression, it is also clear that methylation-dependent regulatory sequences could be found in other parts of the body of the gene or its flanking sequences. Most genome-wide DNA methylation screens focus on promoter regions for reasons of feasibility. By focusing on a short-list of selected genes we were able to examine the entire genomic loci of these genes using a combination of methylated DNA immunoprecipitation (MeDIP) and comprehensive high-density oligonucleotide arrays. Our study provides evidence for association between male physical aggression and differentially methylated regions in cytokines and their transcription factor genomic loci.

## Results

### Associations of Differential DNA Methylation with Physical Aggression in Cytokine Genes

**Figure 1:**
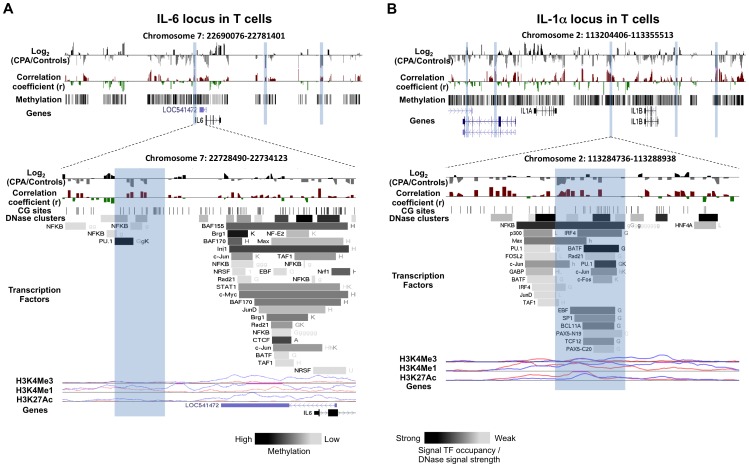
DNA methylation differences between CPA (n = 8) and control (n = 12) groups in pro-inflammatory cytokines IL-6 and IL-1α loci in T cells. Expanded views from the UCSC genome browser of IL-6 (**A**) and IL-1α (**B**) loci located on chromosomes 7 and 2 are depicted (top panel). In both panels, the first track shows the average MeDIP probe log2-fold differences (scale top panel: −0.4 to 0.4, botom panel: −1.0 to 1.0) between chronic physical aggressive (CPA) and control groups for T cells. In black are probes that are more methylated and in gray are those that are less methylated in the CPA group. Highlighted in blue are regions significantly differentially methylated between the groups. The second track shows Pearson’s correlation coefficient values (scale top panel: −0.4 to 0.4, botom panel: −0.7 to 0.7) calculated between the MeDIP microarray probe intensities and the plasma IL-6 (**A**) and IL-1α (**B**) levels obtained from the same subjects (n = 20). In red are probes whose methylation levels correlate positively with the cytokine level in plasma and in green are those that correlated negatively. In the top panel, the lowest track shows the average methylation level for all the subjects in T cells estimated from the microarray data. The bottom panel zooms on the closest region upstream of the TSS where the CPA group is found significantly less methylated than the control group in the IL-6 (**A**) and IL-1α (**B**) loci. In both regions, an overall positive correlation between methylation and cytokine level in plasma is observed but only reached significance after correcting for multiple testing in IL-1α (**B**) differentially methylated region. The regulatory elements from ENCODE identified in these regions (see methods) are shown in the additional tracks. First, shown with black lines, is the location of individual CpG sites. Second, is the location of DNase hypersensitive clusters where black indicate strong signal and grey a weaker signal from ChIP-seq data in 24 cell lines. Third is the location of transcription factors (TF) identified from ChIP-seq data in 24 cell lines where black indicate a strong and grey weaker signal occupancy. The letter next to the TF boxes identified the cell line where it was found enriched (see Table S1 for the full legend). The last tracks, identified the level of enrichment of three histone marks determined from ChIP-seq assay, histone 3 lysine 4 tri- and mono-methylation as well as histone 3 lysine 27 acetylation in two cell lines, GM12878 (pink) and K562 [106].

**Figure 2:**
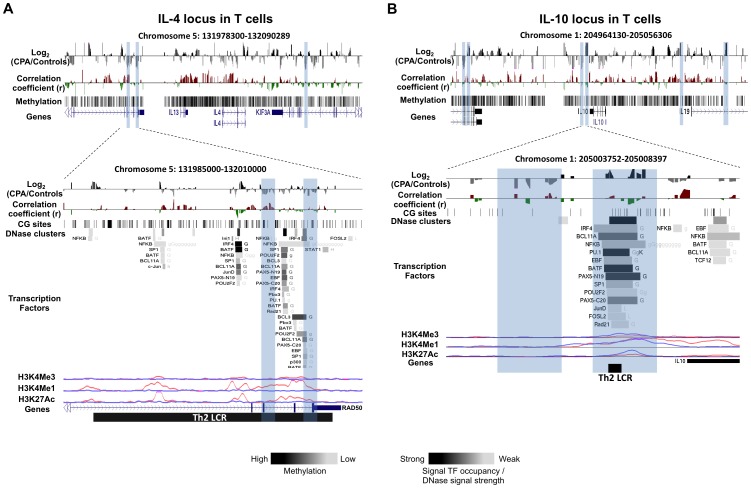
DNA methylation differences between CPA (n = 8) and control (n = 12) groups in anti-inflammatory cytokines IL-4 and IL-10 loci in T cells. Expanded views from the UCSC genome browser of IL-4 (**A**) and IL-10 (**B**) loci located on chromosomes 5 and 1 are depicted (top panel). In both panels, the first track shows the average MeDIP probe log2-fold differences (scale top panel: −0.4 to 0.4, botom panel: −1.0 to 1.0) between chronic physical aggressive (CPA) and control groups are shown for T cells. In black are probes that are more methylated and in gray are those that are less methylated in the CPA group. Highlighted in blue are regions significantly differentially methylated between the groups in T cells for IL-4 (**A**) and IL-10 (**B**). The second track shows Pearson’s correlation coefficient (scale top panel: −0.4 to 0.4, botom panel: −0.7 to 0.7) calculated between the MeDIP microarray probe intensities and the plasma IL-4 (**A**) and IL-10 (**B**) levels obtained from the same subject (n = 20). In red are probes whose methylation levels correlate positively with the cytokine level in plasma and in green are those that correlated negatively. In the top panel, the last track shows the average methylation level for all the subjects in T cells for IL-4 (**A**) and IL-10 (**B**) estimated from the microarray data. The bottom panel shows differentially methylated regions located within known Th2 LCRs where the CPA group is found significantly less methylated than the control group in the IL-4 locus (**A**) and significantly more methylated in the IL-10 locus (**B**). For the first differentially methylated region in IL-4, an overall positive correlation between methylation and IL-4 level in plasma is observed but did not reach significance after correcting for multiple testing (**A**, bottom panel). In the region more methylated in the CPA group of the IL-10 locus (**B**, bottom panel), the overall correlation with IL-10 level in plasma is negative although not significant after corrections for multiple testing. The regulatory elements from ENCODE identified in these regions (see methods) are shown in the additional tracks. First, shown with black lines, is the location of individual CpG sites. Second, is the location of DNase hypersensitive clusters where black indicate strong signal and grey a weaker signal from ChIP-seq data in 24 cell lines. Third is the location of transcription factors (TF) identified from ChIP-seq data in 24 cell lines where black indicate a strong and grey weaker signal occupancy. The letter next to the TF boxes identified the cell line where it was found enriched (see Table S1 for the full legend). The last tracks, identified the level of enrichment of three histone marks determined from ChIP-seq assay, histone 3 lysine 4 tri- and mono-methylation as well as histone 3 lysine 27 acetylation in two cell lines, GM12878 (pink) and K562 [106].

We generated and analyzed DNA methylation profiles covering the genomic loci of five cytokines (IL-1α, IL-6, IL-8, IL-4 and IL-10) and three transcription factors (NFkB, NFAT5 and STAT6) known to regulate these cytokines. Profiles were generated from T cells and monocytes of adult males with normal (control) and chronic physical aggressive (CPA) trajectories from childhood to late adolescence. The five cytokines were selected for profiling because they have been shown to have lower plasma levels in CPA adult males [32]. As describe above, previous studies have shown that WBCs DNA methylation is associated with environmental exposure and correlate with circulating cytokine levels [61], [63], [70]. Since WBCs comprise heterogeneous cell populations, which could mask cell-type specific methylation differences, we isolated and examined CD3+ T cells and monocytes since these cell types express the cytokines and transcription factors that were previously shown to be different in the CPA group. The profiles were generated using MeDIP followed by hybridization to microarrays with probes placed at 100 bp-spacing starting from about −40 Kb before the transcription start site of each gene of interest to about 40 Kb after its transcription end site. Thus, the profiles included not only the promoters and bodies of these genes but also more distant sites in order to identify any genomic site with methylation levels altered in CPA individuals that might explain the previously observed lower cytokine levels. Differences between the CPA and control groups were found throughout each cytokine loci in both cell types analyzed (Table 1). In total, 48 differentially methylated regions associated with CPA (∼1000 bp, log2-fold >0.5, FDR <0.2) were identified in the cytokine loci, 20 in T cells and 28 in monocytes. Surprisingly, none of these were located in the promoters of the candidate cytokines, defined as the region from −2000 bp to +1000 bp of the transcription start site, although one differentially methylated region was found just outside the promoter (−2042 bp) of IL-6 (Figure 1). Two differentially methylated regions were found in the enhancer/insulator locus control region (LCR) of the IL-4 and IL-10 loci (Figure 2). Each cytokine gene loci included several other genes, and some of these non-candidates were in fact closer to several differentially methylated regions than the originally short-listed genes. Tables listing all differentially methylated regions associated with aggression for each cell type along with the gene nearest to the region can be found in Spreadsheet S1.

**Table 1:** Differentially methylated regions between CPA (n = 8) and control (n = 12) groups and correlation with cytokine levels in plasma.

Gene locus	Distance from TSS (kb)	More methylated in		Pearson’s correlation coefficient R (FDR)		Transcription factors (ENCODE)	DNase cluster (ENCODE)
		Monocytes	T cells	Monocytes	T cells		
	−**4**		**Control**			**NFkB, PU.1**	**yes**
**IL-6**	18	CPA	Control			–	yes
	35	Control	Control	0.5 (0.08)		NRSF	yes
	+33.5		CPA			–	yes
	21	Control				Pbx3	yes
	19	Control	Control			PU.1	yes
	7	Control				–	–
	−24	Control		0.45 (0.07)		CEBPB	yes
**IL-1α**	−**26**		**Control**		**0.52 (0.14)**	**NFkB, Max, C-Jun, FOSL2, EBF, SP1, BATF, IRF4, BCL11A, JunD, Pax5, TAF1, TCF12, Rad21, c-Fos, PU.1**	**yes**
	−40	Control				–	–
	−44.5	Control				–	yes
	−46.5	Control				NFkB, EBF, Pbx3, BCL11A, TCF12	yes
	−49.5	CPA				–	–
	−60.5	CPA	Control	−0.58 (0.02)		–	yes
	−81		Control		0.8 (0.1)	–	–
	−40	CPA				–	yes
	−36.5		CPA			c-Myc, BAF155, STAT1, JunD	yes
	−29.5	CPA				–	–
	−27	CPA		−0.53 (0.07)		–	yes
**IL-8**	−**19**	**CPA**				**c-Jun, JunD**	**yes**
	−**16.5**	**CPA**				**–**	**yes**
	8	Control				–	–
	+13.5	CPA				–	yes
	17		Control			–	yes
	32	CPA				PU.1	yes
	36		Control		0.6 (0.06)	c-Jun, GR	yes
	−**35**		**Control**			**NFkB**	**yes**
	**Th2 LCR**						
	−**31.5 Th2 LCR**		**Control**			**NFKB, BCL3, SP1, EBF, IRF4, POU2F2,** **BCL11A, EBF, p300, BATF**	**yes**
	−16.5	Control				PU.1, Rad21, NFkB, CTCF, EBF, BCL3	yes
**IL-4**	+3 (Intron 2)	Control				c-Jun, BAF155, BAF170, Brg1, Ini1, NFkB,CTCF, GR, TAF-1, JunD	yes
	+8 (Intron 3)	Control				NFkB, SREBP2	yes
	+23.5	CPA				SREBP1	–
	27	CPA				–	–
	+31.5		Control			SREBP1	yes
	+34.5	CPA				–	–
	37	Control				–	–
	43		Control			PU.1	yes
	42		CPA			GR	yes
	**7**		**Control**			**–**	**–**
**IL-8**	**6 Th2 LCR**		**CPA**			**Pol2, miRNA, NFkB, ZNF263, IRF4, BCL11A, PU.1, EBF, BATF, PAX5, SP1, POU2F2, JunD, FOSL2, Rd21**	**yes**
	4	CPA				NFkB, EBF, BATF, BCL11A, TCF12, Max, IRF4, BCL3, PAX5, SP1	yes
	−16	Control				BCL3, Pbx3	yes
	−23		CPA			NfkB, BAF170	yes
	−37		CPA			NFkB, CTCF, CEBPB, JunD, RAD21, STAT1	yes

**Figure 3:**
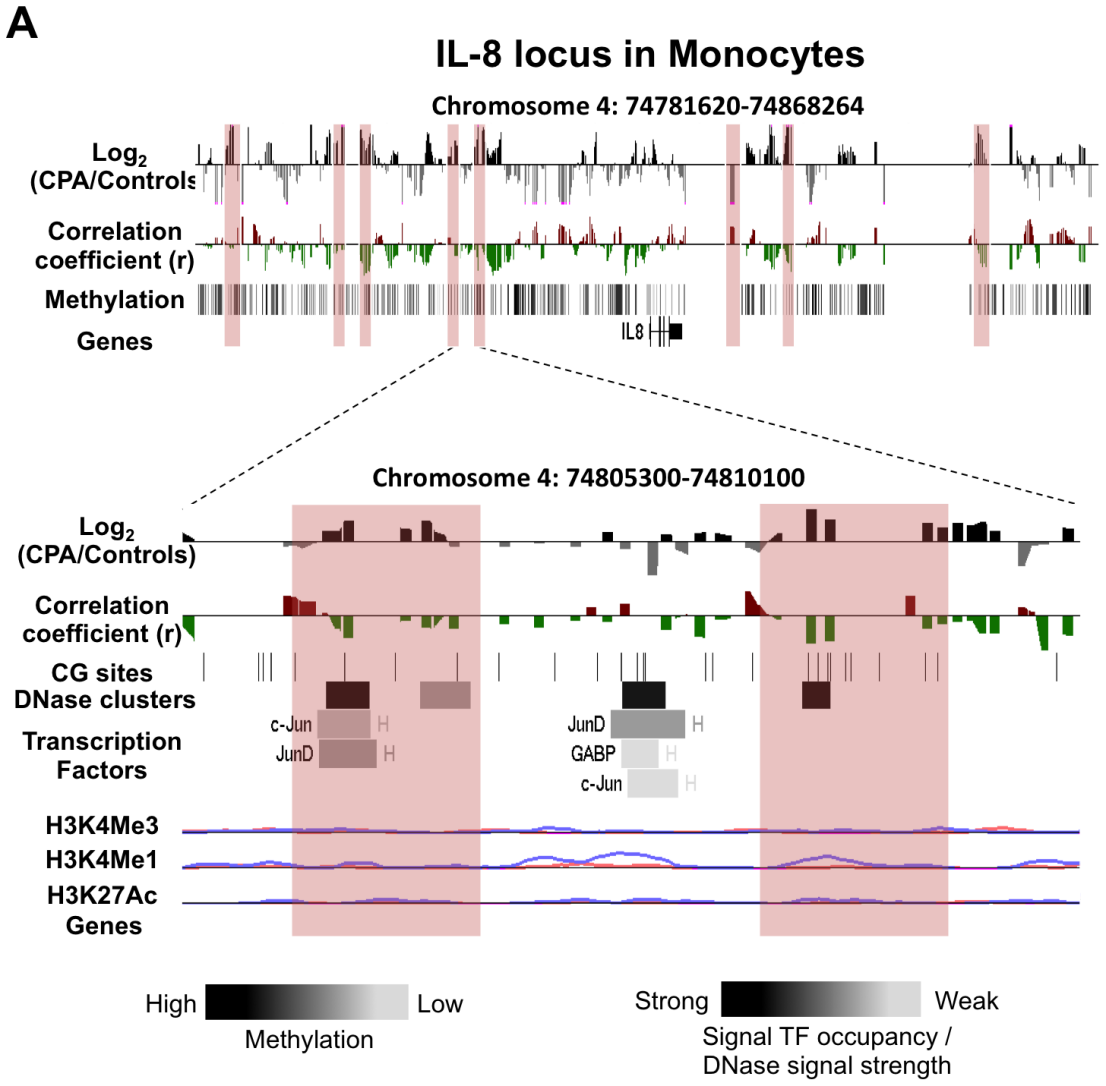
DNA methylation differences between CPA (n = 8) and control (n = 12) groups in pro-inflammatory chemokine IL-8 locus in monocytes. Expanded views from the UCSC genome browser of IL-8 (**A**) locus located on chromosomes 4 is depicted (top panel). In both panels, the first track shows the average MeDIP probe log2-fold differences (scale top panel: −0.4 to 0.4, botom panel: −1.0 to 1.0) between chronic physical aggressive (CPA) and control groups are shown for monocytes. In black are probes that are more methylated and in gray are those that are less methylated in the CPA group. Highlighted in red are regions significantly differentially methylated between the groups. The second track shows Pearson’s correlation coefficient (scale top panel: −0.4 to 0.4, botom panel: −0.5 to 0.5) calculated between MeDIP microarray probe intensities (DNA methylation levels) and the plasma IL-8 levels obtained from the same subject (n = 20). In red are probes whose methylation levels correlate positively with the cytokine levels in plasma and in green are those that correlated negatively. In the top panel, the last track shows the average methylation level for all the subjects in monocytes estimated from the microarray data. The bottom panel shows the closest region upstream of the TSS where the CPA group is found significantly less methylated than the control group in the IL-8 locus. In both regions, an overall negative correlation between methylation and cytokine level in plasma is observed but did not reach significance after correcting for multiple testing. The regulatory elements from ENCODE identified in these regions (see methods) are shown in the additional tracks. First, shown with black lines, is the location of individual CpG sites. Second, is the location of DNase hypersensitive clusters where black indicate strong signal and grey a weaker signal from ChIP-seq data in 24 cell lines. Third is the location of transcription factors (TF) identified from ChIP-seq data in 24 cell lines where black indicate a strong and grey weaker signal occupancy. The letter next to the TF boxes identified the cell line where it was found enriched (see Table S1 for the full legend). The last tracks, identified the level of enrichment of three histone marks determined from ChIP-seq assay, histone 3 lysine 4 tri- and mono-methylation as well as histone 3 lysine 27 acetylation in two cell lines, GM12878 (pink) and K562 [106].

In bold are regions that are illustrated in Figures 1, 2 and 3.

To better understand the regulatory factors potentially affected by the differentially methylated regions associated with aggression, we compared each differentially methylated region against transcription factor binding sites, DNase clusters and histone mark enrichments recorded in the Encyclopedia of DNA Elements (ENCODE) database (http://genome.ucsc.edu/ENCODE/), derived previously from 24 different cell lines (histone mark profiles were available for only 8 of the cell lines). In total, 25 of the 48 differentially methylated regions associated with CPA coincided with transcription factor binding sites (Table 1), and many of the bound transcription factors are known regulators of cytokines such as NFkB, GR, PU.1, STAT and BAFF family members. A high fraction of the differentially methylated regions associated with aggression (35 of 48) coincided with DNase hypersensitive clusters indicating that these regions have an open chromatin state in at least one cell line and therefore likely to play regulatory roles (Table 1). The number of cell lines in which the DNase clusters were observed is shown in Figures 1, 2, 3 as well as in the Figures S1, S2, S3. Histone marks are included in Figures 1, 2, 3 and Figures S1, S2, S3 but only those delineated in cell lines related to T cell and monocytes: a lymphocyte cell line (GM12878), a lymphoblastoid cell line, and K562, an erythroleukemia cell line. In several examples enrichment of particular histone marks coincides with differential methylation associated with CPA. The H3K4Me1 histone mark is mainly associated with enhancers and with DNA regions downstream of transcription start sites whereas H3K4Me3 is mainly associated with promoters and enhancers that are active or poised to be activated [71]. The H3K27Ac mark is thought to enhance transcription possibly by blocking the spread of the repressive histone mark H3K27Me3 [72].

### Associations between Differentially Methylated Regions and Cytokine Levels

Using previously measured cytokine protein levels in the same set of males [32], we were able to test for associations between DNA methylation and cytokine levels. Of the 48 differentially methylated regions associated with aggression, 7 regions were significantly correlated with cytokine levels encoded by the closest genes in blood plasma (FDR <0.2; Table 1). All but two were positively correlated with the cytokine levels in blood.

The differentially methylated region associated with aggression closest to a cytokine transcription start site was located 2042 bp upstream of the pro-inflammatory cytokine IL-6 and was less methylated in the CPA group compared to controls in T cells (Figure 1A and Figure S1). This region contains a DNAse hypersensitive site and a peak of H3K4Me1 in the two lymphocytes cell lines that were studied in the ENCODE project. Interestingly, this region also coincides with the binding sites of transcription factors PU.1 and NFkB. PU.1 is known to act as both a repressor and activator of several cytokine genes [73] and has been proposed to be involved IL-6 expression [74]. Moreover, this IL-6 upstream region has been previously reported to have basic transcriptional activity in gene reporter luciferase assays [75]. A second region less methylated in the T cells of CPA individuals compared to controls was located in the IL-1α locus (Figure 1B and Figure S1B), located 27 Kb upstream of IL-1α. The region is of interest because its methylation levels are significantly correlated with IL-1α levels (R = 0.52; FDR <0.14), and it contains many regulatory elements identified by ENCODE (Figure 1B bottom, highlighted in blue). These include a DNase hypersensitive site, H3K4Me1 and H3K27Ac enrichment peaks and several transcription factor-binding sites, including those of both activators and repressors. Transcription factors BATF, NFkB and PU.1 have the strongest binding signal in this region.

In the locus of IL-4, we identified ten regions that were differentially methylated between CPA and controls, three of these regions were found in T cells. Two of these regions that are less methylated in the T cells of CPA individuals, contain several ENCODE regulatory elements (Figure 2A bottom, Figure S2A), including a DNase hypersensitive cluster, enrichment for histone marks H3K4Me3 and H3K27Ac, and several transcription factor binding sites (Figure 2A bottom, highlighted in blue). Theses regions are located in the Th2 cytokine Locus Control Region (LCR) known to regulate the expression of IL-4 locus.

Three differentially methylated regions associated with aggression lie within 10 Kb downstream of the IL-10 transcription start site. Two regions are differentially methylated in T cells the first (+6 Kb) is more methylated in CPA individuals whereas the second (+7 Kb) is less methylated. The third difference lies in the 3′UTR of IL-10 and is more methylated in monocytes of CPA individuals (Figure 2B and Figure S2B). Only the first region (+6 Kb) contains several of the ENCODE regulatory elements (Figure 2B bottom, highlighted in blue), including a strong DNase hypersensitive cluster, enrichment for histone marks and several transcription factor binding sites. Interestingly, this region was previously found to have promoter/enhancer activity in Th2 cells and was proposed to function as a LCR for the IL-10 gene locus [76].

The IL-8 locus contains eight regions differentially methylated between CPA and controls in monocytes and three in T cells (Figure 3A and Figure S3). Since IL-8 is mainly expressed in monocytes, we focused on two regions more methylated in monocytes of CPA individuals that are 16 Kb and 19 Kb upstream of IL-8, respectively (Figure 3A bottom, highlighted in red). Both regions, though located far from the gene promoter, were found to include a strong DNase hypersensitive cluster, and the second region contained a small H3K4Me1 peak in the K652 cell lines. Interestingly, binding sites of transcriptional activators c-Jun and JunD were identified in the first region. C-Jun and JunD are both part of the AP-1 transcriptional complex, a well-known activator of many genes including cytokines [77].

### Associations of DNA Methylation with Physical Aggression in the Loci of Transcriptional Regulators of Differentially Expressed Cytokines

The microarray used in this study included the genomic loci of three transcription factors, NFkB1, NFAT5 and STAT6 that are classical regulators of the cytokines analyzed above. Differentially methylated regions between the CPA and control groups were identified throughout the loci in both cell types analyzed (Table 2) with both increased and decreased methylation levels in CPA individuals compared to controls. Each locus had at least ten differentially methylated regions (∼1000 bp, log2-fold >0.5, FDR <0.2). Similarly to the cytokine loci, the locations of some differentially methylated regions associated with aggression coincided with the location of regulatory regions such as the promoters (as defined earlier −2000 bp to 1000 bp of TSS) of both STAT6 isoforms as well as in the NFkB1 short transcript (p50) that were more methylated in the CPA group (Table 2). We found 37 differentially methylated regions associated with CPA within the NFkB locus, 21 of these regions were located within the gene body (Figure S4). In the NFAT5 locus, 19 differentially methylated regions between CPA and controls were identified and all of them except one were located within the body of the gene (Figure S5). A total of 10 regions were identified in STAT6 locus, two of the differentially methylated regions associated with CPA were found in T cells and all other differentially methylated regions associated with CPA in STAT6 were found in monocytes (Figure S6).

**Table 2:** Differentially methylated regions between CPA (n = 8) and control (n = 12) groups identified in the loci of three transcription factors known to regulate cytokines.

Gene locus	Distance from TSS (kb)	More methylated in		Transcription factors (ENCODE)	DNase clusters (ENCODE)
		Monocytes	T cells		
	−32 p150		Control		yes
	−8 p150	CPA		PU.1	yes
	+6.5 p150	CPA		–	–
	+10 p150		CPA	CEBPB	yes
	+12 p150	CPA		–	–
	+24 p150	CPA		–	yes
	+26 p150		Control	BATF, NFkB, EBF	yes
	+30.5 p150	CPA	Control	–	yes
**NFKB1** Two transcripts: p150and p50	+34 p150, −42 p50	CPA		JunD, IRF4, BCL11A, NFkB, BATF,POU2F2, PAX5, C-Jun	yes
	+36 p150, −40 p50	CPA		–	yes
	+39 p150, −37 p50	CPA		–	–
	+53 p150, −21 p50	CPA		–	yes
	+65 p150, −9 p50	CPA		NFkB	yes
	+76 p150,		Control	–	–
	TSS p50				
	+80 p150,	CPA	Control	–	yes
	+4 p50				
	+8 p50	CPA		IRF4, BAF, EBF, JunD	yes
	+11.5 p50	Control		–	yes
	+20 p50		Control	NFkB	yes
	+26 p50	Control		–	–
	+31 p50		CPA	–	–
	+35 p50	CPA		–	yes
	+37 p50		CPA	–	yes
	+45 p50	CPA		FOSL2, HNF4A, CTCF, USF-1	yes
	+53 p50		CPA	GR	yes
	+55 p50	CPA		–	–
	+68 p50	Control		–	–
	+83 p50	Control	Control	–	–
	−23	Control		C-Jun, PAX3, JunD, NFkB	yes
	9	CPA		–	–
	22		CPA	–	–
	+25.5	CPA	CPA	–	yes
	43	CPA		–	yes
	52	Control		CEBPB	
	53	CPA		–	yes
	54		Control	–	yes
	55	CPA	CPA	–	–
**NFAT5**	62		Control	–	–
	67	CPA		–	–
	77	CPA	Control	–	–
	84	Control		Ini1	–
	85	CPA	Control	–	–
	87	CPA	Control	–	–
	91	CPA	Control	–	–
	112	CPA	Control	–	–
	128	CPA		–	–
	136	CPA		–	–
	+53 isoform 2	CPA		NFkB	yes
	+25 isoform 2	CPA		HEY1, Brg1, TAF1, C-Myc, NFkB, STAT2, Max, BAF155, Ini1, STAT1, Pu.1, BAF170	yes
	+20 isoform 2	Control		Ini1	yes
	+6 exon 8 to 10 isoform 2	Control		–	yes
	+3 exon 1 isoform 2	Control		–	yes
					
**STAT6** Two transcripts: isoform 1 and isoform 2	**TSS isoform 2, +18 isoform 1**		**CPA**	**C-Myc, max, NFkB, brg1, BAF170, STAT1, BAF155, TAF1, Ini1, P1, POUF2L2, BCL3, PU.1, IRF4, PAX5, HEY1, CEBPB, EBF, TCF12, NRSF**	**yes**
	−**1.5 isoform 1**		**CPA**	**HNF4, CEBPB, FOSL2, RXRA, p300, BHLHE40, JunD, ERRA, USF-1, NFkB**	**yes**
	−10 isoform 1	Control		HNF4, CEBPB, FOSL2, BAF155, p300, HEY1, RXRA, JunD	yes
	−14 isoform 1	Control		–	yes
	−23 isoform 1	Control		–	yes

**Figure 4:**
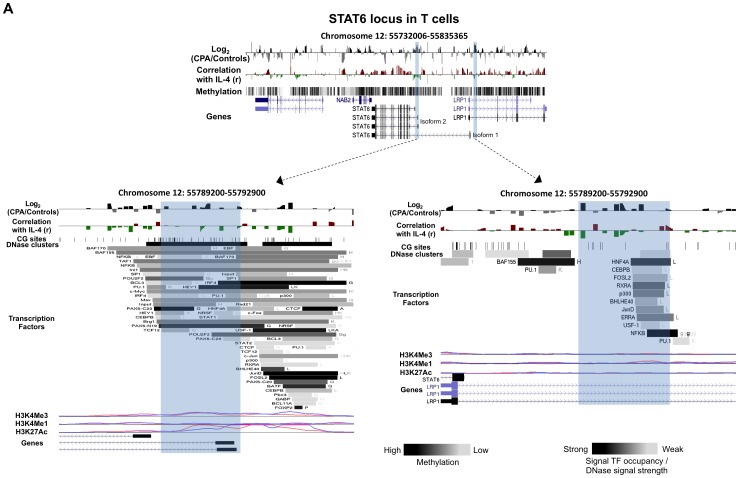
DNA methylation differences between CPA (n = 8) and control (n = 12) groups in cytokine’s transcription factor STAT6 in T cells. Expanded views from the UCSC genome browser of STAT6 (**A**) locus located on chromosomes 12 is depicted (top panel). In both panels, the first track shows the average MeDIP probe log2-fold differences (scale top panel: −0.4 to 0.4, botom panel: −1.0 to 1.0) between chronic physical aggressive (CPA) and control groups for T cells. In black are probes that are more methylated and in gray are those that are less methylated in the CPA group. Highlighted in blue are regions significantly differentially methylated between the groups. The second track shows Pearson’s correlation coefficient (scale top panel: −0.4 to 0.4, botom panel: −0.6 to 0.6) calculated between MeDIP microarray probe intensities and the plasma IL-4 (first) and IL-10 (second) levels obtained from the same subject. These correlations did not reach significance after correcting for multiple testing in T cells. In red are probes whose methylation levels correlate positively with the cytokine level in plasma and in green are those that correlated negatively. In the top panel, the last track shows the average methylation level for all the subjects in T cells estimated from the microarray data (n = 20). The bottom panel shows the two regions close to the TSS of each STAT6 isoform where the CPA group is found significantly more methylated than the control group. The regulatory elements from ENCODE identified in these regions (see methods) are shown in the additional tracks. First, shown with black lines, is the location of individual CpG sites. Second, is the location of DNase hypersensitive clusters where black indicate strong signal and grey a weaker signal from ChIP-seq data in 24 cell lines. Third is the location of transcription factors (TF) identified from ChIP-seq data in 24 cell lines where black indicate a strong presence and grey weaker signal occupancy. The letter next to the TF boxes identified the cell line where it was found enriched (see Table S1 for the full legend). The last tracks, identified the level of enrichment of three histone marks determined from ChIP-seq assay, histone 3 lysine 4 tri- and mono-methylation as well as histone 3 lysine 27 acetylation in two cell lines, GM12878 (pink) and K562 [106].

In bold are regions that are illustrated in Figures 4.

As we did for the cytokine genes, we examined whether the differentially methylated regions associated with aggression contained previously published transcription factor (TF) binding sites, DNase hypersensitive clusters and enrichment for histone modifications H3K4Me1, H3K4Me3 and H3K27Ac using the ENCODE database (http://genome.ucsc.edu/ENCODE/). We found that most of the differentially methylated regions associated with CPA identified in NFkB1 and STAT6 loci in both cell types were located within DNase hypersensitive clusters, transcription factors binding sites and H3K4Me1, H3K4Me3 and H3K27Ac peaks, supporting a regulatory role for these regions (Table 2 and Figures S4 and S6). In contrast, only a few differentially methylated regions between CPA and controls in NFAT5 locus were located within regulatory elements. None of the regions were located within CpG islands.

The methylation profile of the STAT6 locus in T cells is illustrated in Figure 4. Two regions in this locus are more methylated in the T cells of CPA individuals compared to controls (Figure 4A and Figure S6, highlighted in blue). The first region is located at the transcription start site of the STAT6 isoform 2 gene (Figure 4 left bottom panel), and the second region is located 1 Kb upstream of STAT6 isoform 1 (Figure 4 right bottom panel). Both regions coincide with DNase hypersensitive clusters and many transcription factor binding sites such as IRF4, HEY1 and NFkB. Enrichment for H3K4Me3 and H3K27Ac marks were found in the first region (TSS isoform 1) in both cell lines whereas only a small peak of H3K4Me1 enrichment was found in the second region in the GM12878 cell line.

**Figure 5:**
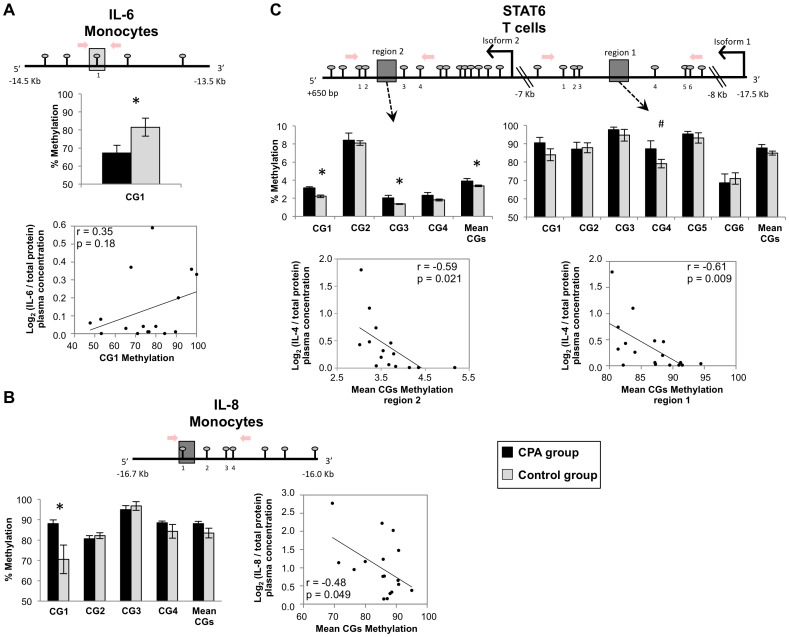
Pyrosequencing analyses of differentially methylated sequences in T cells and monocytes between CPA (n = 8) and control (n = 12) groups. DNA methylation differences (%) between the aggressive groups in the IL-6 (**A**), IL-8 (**B**) and STAT6 (**C**) genes as determined by pyrosequencing. CpG sites near the significant probes were analyzed in triplicate. For each gene, the mean methylation per group per CpG with the standard error of the mean (SEM) are shown in the bar graph (top panel). The rightmost bar indicates the mean methylation levels of all CpG sites analyzed in the region. A map of the sites relative to the transcription start site is shown above the bar graph. Each line with a circle represents a CpG site. The location of probes which fold difference is significantly different between the groups is identified by a grey square where dark and light grey indicated more or less methylated in the CPA group respectively. Red arrows delimit the region analyzed by pyrosequencing. Correlations between mean methylation levels obtained by pyrosequencing for each gene and expression levels in plasma obtained by ELISA for each cytokine [32] is shown in the bottom panel. Correlation statistics were performed using Pearson correlation. All error bars represent standard error of the mean (SEM). The symbol ‘#’ denotes a p-value ≤0.1 and the symbol ‘*’ denotes a p-value ≤0.05 as determined by a Student *t*-test.

We have further confirmed the methylation differences between CPA and control groups and correlation with the cytokine levels in plasma in regions within the IL-6, IL-8 and STAT6 loci by pyrosequencing (Figure 5A, 5B and 5C; see Methods). Intriguingly, we note that the pyrosequencing methylation levels in *STAT6* gene are significantly associated with IL-4 levels in plasma.

## Discussion

Several lines of evidence suggest that cytokines are associated with animal and human aggression [78]–[80] and IL-6 was causally linked to aggression in mice by gene knockout evidence [31]. Our analyses of males whose level of aggression were followed for a 22 year period revealed an association between cytokine levels in plasma and chronic physical aggression: young adult men with a history of chronic physical aggression during childhood have lower baseline concentrations of two pro (IL-1α, IL-6 and IL-8) and two anti (IL-4 and IL-10) -inflammatory cytokines than comparable young adult men without a history of chronic physical aggression during childhood [32]. Here we show data that is consistent with the hypothesis that DNA methylation could be part of the mechanisms responsible for differences in cytokine levels in the chronic aggression group. Indeed, we found differentially methylated regions to associate with CPA in both the cytokine loci as well as in their transcription factors loci analyzed. Some of these differentially methylated regions found in IL-6, IL-4, IL-10, STAT6 and NFkB were located in known regulatory regions whereas others, to our knowledge, were previously unknown as regulatory areas. However, using the ENCODE database, we were able to identify key regulatory elements in many of these regions that indicate that they might be involved in the regulation of cytokine expression. Further work will be needed to investigate the exact role of these new candidate regulatory regions in the regulation of transcription of these genes and particularly in the context of problems with regulation of physical aggression during childhood.

Interestingly, four of our differentially methylated regions associated with aggression were located within known cytokines regulatory regions. Particularly, three regions lie within Th2 cytokines LCRs, two in the IL-4 loci and one in the IL-10 loci. These LCRs have been shown to have enhancer/insulator activity in Th2 cell differentiation [44], [76], [81]. Changes in chromatin remodeling and DNA methylation in this region are involved in regulating expression of IL-4 and IL-13 during Th2 differentiation [44], [81]. It was previously proposed that DNA demethylation is required for the maintenance of IL-4 LCR enhancer activity in Th2 differentiated cells [81]. STAT6 transcription factor was also shown to be required for the remodeling of this LCR during Th2 cell differentiation [81]. We observed in the same region lower methylation in the CPA individuals in T cells and overall lower IL-4 plasma levels compared to controls (Figure 2A). Since we did not examine the methylation levels in specific T cells subpopulations, we don’t know what this demethylation in CPA individuals means in term of Th2 LCR activity. The fact that it occurs in this important regulatory region suggests however that the activity of the IL-4 locus might be altered in individuals with history of chronic physical aggression during childhood. On the other end, we found higher methylation in the promoter of STAT6 genes in the CPA individuals (Table 2). STAT6 is a well-known regulator of IL-4 in T cells, specifically in Th2 differentiating cells. As mentioned earlier, it can induce expression of IL-4 by interacting with the Th2 LCR [81] but also by activating the expression of the transcription factor GATA-3 that bind directly to the IL-4 promoter [82]. The methylation differences observed in the promoter of isoform 2 as well as in intron 1 of isoform 1 were validated by pyrosequencing. Intriguingly, we note that the pyrosequencing methylation levels in *STAT6* gene are significantly associated with IL-4 levels in plasma, further supporting a regulatory role for these sites in regulation of IL-4 by STAT6. This is consistent with the hypothesis that increased methylation leads to a decrease in STAT6 expression and that could lead to reduced activation of IL-4 locus and lower levels in plasma.

DNA methylation analyses were not previously performed for the LCR in IL-10 locus but it stands to reason that as the LCR in IL-4 locus, DNA demethylation is needed for the maintenance of this LCR functionality. The LCR in IL-10 locus was found more methylated in the CPA individuals compared to controls. These results are consistent with the hypothesis that higher methylation in T cells in this region may account, in part, for the decreased in IL-10 expression observed in the plasma of the CPA group. Similarly, IL-8 results are consistent with the hypothesis that higher methylation in CPA individuals reduces IL-8 expression levels by inhibiting binding of transcriptional activators.

In, IL-1α, IL-6 and IL-4 LCRs our study delineated regions with lower methylation in T cells in CPA individuals who also show lower levels of these cytokines in plasma (Figure 1A, 1B and 2A bottom, highlighted in blue). These results might be consistent with the hypothesis that lower methylation in these regions permits more frequent binding of a putative repressor leading to the decreased IL-4, IL-6 and IL-1α expression. Indeed, there is evidence that increased DNA methylation of certain regions is associated with increased gene expression. For example, DNA methylation in a repressor-binding site of the multidrug-resistance gene (MDR-1) enhancer was shown to increase its expression [83]. Another example is the H19/IGF2R imprinted control regions (ICR). Methylation of the ICR on the paternal allele prevents H19 activity but allows IGF2 activity whereas hypomethylation on the maternal allele allows H19 activity but prevents IGF2 activity [84]. These hypotheses on the effect of DNA methylation on cytokine genes expression need to be tested *in vitro* as well as *in vivo* as the data presented here only reveal associations but do not provide causal relationships between the differentially methylated regions and expression.

Our results also point to the value of locus-wide analysis of DNA methylation. Most studies are limited to promoter regions. Had we used this approach here we would have missed most or all of the differentially methylated regions that we had identified in this study. Many of which contain high concentration of regulatory signals. This suggests that many of the published failures in identification of differentially methylated regulatory regions might be a consequence of limiting the analyses to known promoter regions.

Taken together the fact that a high fraction of the differentially methylated regions between the CPA and control groups were localized in either known regulatory regions or regions that were independently shown to contain a high concentration of regulatory chromatin marks, DNase hypersensitivity and transcription factors, supports the hypothesis that they are involved in differential regulation of cytokine expression between CPA and control groups. Indeed, several methylated regions located within the cytokine loci were found to significantly correlate with cytokine levels in plasma.

Although our list of differentially methylated regions associated with CPA is comprehensive it is still possible that additional differentially methylated regulatory regions exist in these loci that were not discovered in our analysis. The small sample size could result in loss of power to detect additional significant correlations. In addition, the associations of differentially methylated regions and cytokine expression delineated here are probably an underestimation since we have used plasma cytokine levels rather than transcription rates as a measure of cytokine expression. There are many steps in between initiation of transcription at the promoter and cytokine levels in plasma. Unfortunately, the study design did not allow us to isolate high quality RNA because it was difficult to request that the subjects come to the lab for their blood perfusion, particularly with the chronic aggression group, resulting in a delay between cell harvest and RNA processing. In addition, DNA methylation and expression are fundamentally different measures of cell functional states. DNA methylation levels directly correspond to the number of cells with a methylated/unmethylated cytosine (as a state of methylation of an allele in a particular site is a binary count, in a given cell a site is either methylated or not) whereas protein and mRNA levels are analog measures that average the number of mRNA and protein molecules produced by the cell population. A low level of mRNA or protein measure could indicate either high level of expression in a few cells or low level of expression in many cells.

Several methodological considerations arise in our study. First, with blood available only in adulthood, we cannot establish when during development chronic physical aggression became associated with these particular DNA methylation alterations and cytokine level reduction in plasma. Based on the longitudinal data obtained from this cohort, we can expect that DNA methylation changes appear early in life as chronic physical aggression trajectories usually start in infancy and are associated with numerous factors during the prenatal and early postnatal periods [5]. However, it stands to reason that there is a developmental trajectory of these DNA methylation changes and that changes seen in adults will not be identical to the early changes seen in aggressive children. The second issue relates to cause and effect. We have demonstrated here a highly significant association between DNA methylation alterations and CPA. These changes could be either a cause or an effect of CPA life style. The issue of causality is particularly difficult in human studies although a parallel longitudinal analysis of behavioral and DNA methylation changes could have established at least the temporal relationship between differential DNA methylation of cytokines and aggressive behavior. In addition, childhood abuse or neglect is also known to increase the risk of aggression in adolescents and adults [85] and was also found to associate with DNA methylation differences [59]. Therefore, it is possible that child abuse acts as a third factor in explaining the reported association between aggression and DNA methylation.

Neuroendocrine-immunological abnormalities that are established during a stressful childhood are thought to mediate the development of the pro-inflammatory phenotype in adulthood [33], [34] including reduced cortisol awakening response [86]. This could result in altered expression of certain cytokine levels in plasma, since cortisol levels are known to regulate immune and inflammatory responses. For example, stress has been shown to promote the expression of certain cytokines [87], correlations were found between reduced cortisol and increased aggression in adolescents and young men [88]–[90], and maltreatment in childhood was shown to lead to low basal cortisol in association with conduct and aggressive disorders [91]. However, the associations between stress, cortisol, cytokines and aggression are complex since opposite results have also been reported. Indeed, glucocorticoids produced by the adrenal gland are strong immunosupressors known to directly repress the expression of many cytokines in leucocytes including the cytokines examined here. For example, a study of the males from the present study during adolescence, reported that high cortisol levels were associated with high levels of aggression [92]. Therefore, both hyper- and hypo-active stress responses have been shown to associate with aggression [93].

Unfortunately, although our cohort was longitudinal and behavioral data was longitudinally collected from childhood, DNA from blood cells were collected only once the participants in the study reached adulthood. Future longitudinal studies should address these questions. Second, the results are based on a small sample of chronic aggressive subjects. The two longitudinal studies we used to recruit our subjects from had followed more than 1 000 males from childhood to adolescence. Young adult Caucasian males with a history of chronic physical aggression during childhood are relatively rare [11] and even more difficult to recruit for biological sampling. Thus, the limited numbers are an inherent property of the trait that we are examining. However replications of the present study with other longitudinal samples are obviously needed and should address this issue in part although we don’t expect a large number of CPA subjects in these future studies as well. In any case, the subgroup that was available for the DNA methylation analysis did not differ in its basic behavioral and socioeconomic characteristics from the original group (Table 3). Third, although we ruled out several of the most common confounders, there are some confounders that are tightly correlated with aggression. Cytokine levels as well as DNA methylation were previously associated with psychiatric diseases such as major depression [94], nevertheless the CPA and control groups were not significantly different in levels of anxiety and presence of psychiatric diagnoses as well as age. However, the CPA and control groups were significantly different with respect to variables that are also known to be strongly associated with chronic physical aggression trajectories from childhood to adolescence: childhood hyperactivity and opposition and adulthood criminal behavior [6], [11]. Thus, it is impossible to determine whether the differential DNA methylated regions that we identified associate with aggressiveness or hyperactivity and criminal behavior. This however might be reflecting the simple fact that these behaviors are molecularly and functionally linked within the same biological pathways.

**Table 3:** Characteristics of the chronic physical aggression (CPA) group and control group in the original sample (1) and the selected sample (2) for the methylation analysis.

Variables	Control				CPA				Group effect	
	Mean ± SD or % (n)				Mean ± SD or % (n)					
	Sample 1		Sample 2		Sample 1		Sample 2		Sample 1	Sample 2
Age at blood perfusion	25.64±2.08	*(25)*	25.4±2.71	*(12)*	25.28±2.75	*(7)*	25.8±2.87	*(8)*	*t*(30) = 0.37, *P = *0.71	*t*(18) = −0.26, *P = *0.80
Familial adversity 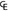	0.34±0.30	*(25)*	0.34±0.29	*(12)*	0.44±0.42	*(7)*	0.51±0.41	*(7)*	*t*(29) = −0.73, *P = *0.47	*t*(17) = −1.06, P = 0.31
Psychiatric record (21 years)	33%	*(7/21)*	60%	*(6/10)*	43%	*(3/7)*	43%	*(3/7)*	*F* exact, 2 tailled: 0.67	*F* exact, 2 tailled: 0.64
Criminal record (21 years)	20%	*(5/25)*	17%	*(2/12)*	71%	*(5/7)*	75%	*(6/8)*	*F* exact, 2 tailled: *0.02*	*F* exact, 2 tailled: *0.019*
Self reported violence (21 years)	10%	*(2/20)*	10%	*(1/10)*	57%	*(4/7)*	57%	*(4/7)*	*F* exact, 2 tailled: *0.02*	*F* exact, 2 tailled: 0.10
Hyperactivity (6 to 15 years)	1.20±0.96	*(25)*	0.91±1.03	*(12)*	2.43±1.27	*(7)*	2.47±1.20	*(8)*	*t*(30) = −2.79, *P = 0.009*	*t(18) = *−*3.11, P = 0.006*
Oppositional disorder (6 to 15 years)	2.16±1.86	*(25)*	2.19±1.92	*(12)*	8.43±7.00	*(7)*	8.14±6.71	*(8)*	*t*(6.2) = −2.35, *P = *0.06	*t(18) = *−*2.93, P = 0.009*
Anxiety (6 to 15 years)	3.36±1.93	*(25)*	3.35±1.81	*(12)*	3.43±1.51	*(7)*	3.48±1.45	*(8)*	*t*(30) = −0.09, *P = *0.93	*t*(18) = −1.62, P = 0.87
Attention deficit (6 to 15 years)	3.2±2.22	*(25)*	3.23±2.18	*(12)*	4.00±2.00	*(7)*	4.00±1.89	*(8)*	*t*(30) = −0.86, *P = *0.40	*t*(18) = −0.81, P = 0.43

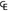
Include mother and father occupational score, familial status (monoparental vs biparental), mother and father age at birth of first child and the years of schooling of the mother and father.

### Conclusions

This study has several implications. Taken together the data is consistent with the hypothesis that cytokines are involved in chronic physical aggression, hence that a peripheral immune component may play a key role in regulating these behavioral states. The results suggest that in addition to differential methylation of critical regions within the cytokine gene loci, differential DNA methylation states of transcription factors that regulate these cytokine genes could be one of the mechanism that explain the decrease in plasma cytokines expression observed in the CPA group.

## Materials and Methods

### Participants

The subjects for the plasma cytokine level analysis (sample 1) [32] were recruited from male participants in two longitudinal studies of child development [15], [95] which identified four different trajectories of physical aggression from age 6 to 15 years: Males in the first trajectory showed very low or no physical aggression at any time-point (low-never); males in the second trajectory started with low-to-moderate rates of physical aggression but declined over time (moderate level desister); those in the third trajectory began with high rates of physical aggression but subsequently declined between ages 12 and 15 (high level desister); males on the fourth trajectory showed consistently high physical aggression from age 6 to 15 years (chronic physical aggression, CPA).

The two groups of Caucasian males we recruited from these longitudinal studies were all born in families with a low socioeconomic status and were living within 200 km from our laboratory at the time of recruitment. The first group was recruited from the chronic physical aggression trajectory (chronic physical aggression group, CPA, n = 8). The control group was recruited from the other three developmental trajectories (Control group, CG, n = 25). Quantification of 10 cytokine levels in plasma was done by ELISA for these subjects (sample 1) using the Quantibody® Human Inflammation Array 1 from RayBiotech (Norcross, United States). The ELISA results showed that compared to the control group, the CPA group had significantly lower levels in plasma of five cytokines; lower pro-inflammatory interleukins IL-1α (*T*(28.7) = 3.48, *P = *0.002) and IL-6 (*T*(26.9) = 3.76, *P = *0.001), lower anti-inflammatory interleukin IL-4 (*T*(27.1) = 4.91, *P = *0.00004) and IL-10 (*T*(29.8) = 2.84, *P = *0.008) and lower chemokine IL-8 (*T*(26) = 3.69, *P = *0.001) (see [32] for details). From these subjects (sample 1), the methylation analysis of these cytokine gene loci and their transcription factors was done on all the 8 subjects from the CPA group and a representative subgroup of 12 subjects was selected for the control group (sample 2). *Note that one CPA subject was removed from the ELISA analysis due to cytokine concentrations outside of the expected range for the standard curve but was included in the methylation analysis.* A schematic overview of the recruitment is illustrated in Figure S7. Characteristics of the original samples (n = 32 sample 1) and the selected subgroups (n = 20 sample 2) are presented in Table 3.

### Ethics Statement

After complete description of the study to the subject, all participants provided written informed consent. The study was carried out in accordance with the Declaration of Helsinki, and was approved by the research ethics committee of the University of Montreal pediatric hospital (St-Justine Hospital).

### Assessment of Subjects’ Familial Adversity, Behavior Problems, Psychiatric Diagnoses and Criminal Records

#### Familial adversity

The index of family adversity is a composite score of the degree of adversity in families ranging from 0 to 1, which has been used regularly with these cohorts. The index includes parent’s level of education, type of employment, age at the birth of their first child and marital status when the subjects were age 6 [16], [96].

#### Physical aggression and other behavior problems

In the course of the longitudinal studies, teachers annually rated the frequency of boys’ physical aggression, opposition, hyperactivity, inattention and anxiety from kindergarten to secondary school with the Social Behavior Questionnaire [96]. The physical aggression ratings were used to trace the developmental trajectories and create the CPA group and the control group (see [6] and [11] for details of the trajectory analyses).

#### Self reported violence

During the data collection at 21 years, subjects were asked how often in the past year they had been implicated in physical fights and how often they had physically attacked someone.

#### Criminal record

Canadian youth between 13 and 17 years who commit delinquent acts are referred to the juvenile courts. Subjects who were found guilty by a juvenile court were identified from official records. Using official records we also identified subjects in each group who had been convicted of a criminal offence between 18 and 24 years.

#### Mental disorders

When the subjects were 15 years, structured psychiatric interviews using a French translation of the Diagnostic Interview Schedule for Children-2.25 (DISC-2.25) [97] were used with the mothers and the subjects to estimate the prevalence of DSM-III-R diagnoses such as: simple phobia, anxiety of separation, generalized anxiety, hyper anxiety, major depression, dysthymia, oppositional disorder and conduct disorder, over the previous 6 months [87]. Subjects were also asked whether they had a psychiatric record during the interview at 21 years.

### CD3+ T cells and Monocytes DNA Preparation

For the study, 20 ml of blood were drawn in EDTA coated-tubes for each subjects. PBMC (whole mononuclear cells from peripheral blood) and T cell isolation procedures were adapted from Current Protocols in Immunology (1997, sections 7.1 and 7.5.1–7.5.11). Briefly, PBMC isolation was done by centrifugation with Ficoll-Paque (GE healthcare, Mississauga, Canada) and washed twice with HBSS (Hanks balanced salt solution, GIBCO, Burlington, Canada). T cells were isolated from the PBMCs by immunomagnetic isolation using CD3 dynabeads (Invitrogen (Dynal Biotech), Burlington, Canada). The beads were washed 3 times and incubated with the PBMCs for 45 min on a rotator at 4°C. Coated CD3+ cells with the dynabeads were isolated using a strong magnet (Steam Cell Technology) and washed 5 times with PBS/FBS. CD3+ cells coated with the dynabeads were then frozen at −80°C until DNA extraction. To isolate monocyte cells, B cells were extracted away from the remaining PBMC with CD9 dynabeads (Invitrogen (Dynal Biotech), Burlington, Canada) following the same procedure as for T cells isolation and then frozen at −80°C until DNA extraction. Monocytes and T cells DNA was extracted with Wizard® Genomic DNA Purification kit (Promega, Madison, United States) following the manufacturer’s protocol.

### Methylated DNA Immunoprecipitation (MeDIP), Amplification and Labeling

The MeDIP analysis was adapted from [98]. Briefly, 2 µg of each of the T cells and monocytes DNA were sonicated and methylated DNA was immunoprecipitated with 10 µg of anti-5methylCytosine (Calbiochem, Billerica, United States). Prior to sonication, two control plasmids were added to the DNA (6ρg each), an unmethylated GFP plasmid and an *in vitro* methylated Luciferase plasmid. The DNA-antibody complex was immunoprecipitated with 5 mg protein A and the methylated DNA was eluted with 150 µl of TE at 1.5% SDS. The input and bound fractions were then purified and validated by PCR analysis for two control genes, H19 (methylated control) and β-actin (unmethylated control) and the two added plasmid, GFP (unmethylated control) and Luciferase (*in vitro* methylated control) using the following primers: H19: forward (5′-TTGGTGGAACACGCTGTGATCA-3′), reverse (5′-GAGCCGCACCAGGTCTTCAG-3′); β-actin: forward (5′-AGCCATAAAAGGCAACTTTCG-3′), reverse (5′-CCAACGCCAAAACTCTCCC-3′); GFP: forward (CCAACGCCAAAACTCTCCC), reverse (AGCCATAAAAGGCAACTTTCG); Luciferase: forward (5′-AGAGATACGCCCTGGTTCC-3′), reverse (5′-CCAACACCGGCATAAAGAA-3′). The input and bound fractions were then amplified in triplicate using the Whole Genome Amplification kit (Sigma, Oakville, Canada). The amplified input and bound fractions were labeled for microarray hybridization with either Cy3-dUTP or Cy5-dUTP (Perkin Elmer, Montreal, Canada) respectively using the CGH labeling kit (Invitrogen, Burlington, Canada) following the manufacturer’s instructions.

### Microarray Design, Hybridization, Scanning and Analysis

A detailed description of the methods and analyses concerning microarrays used in this study were previously described [60]. Briefly, custom 8×44 K tilling arrays (Agilent technologies) containing probes selected to tile all the cytokines and TF loci (∼100 Kb) were manufactured by Agilent (Mississauga, Canada). Three replicate microarrays were hybridized and analyzed for each sample. Extracted probe intensities were analyzed using the R software environment for statistical computing [99]. Log-ratios of the bound (Cy5) and input (Cy3) microarray channel intensities were computed for each microarray and then microarrays were normalized to one another using quantile-normalization [100] under the assumption that all samples have identical overall methylation levels.

Differential methylation between groups of samples was determined at both the probe and region levels (1000 bp regions) to ensure both statistical significance and biological relevance as previously described [60]. At the probe level, a modified t-statistic was computed for each probe corresponding to probe log-ratio differences between CPA and control groups using the ‘limma’ package [101] of Bioconductor [102]. Then, region-level methylation differences were calculated as enrichment of large positive or negative t-statistic values among the probes in each 1000 bp region partition of the loci tiled with probes using the Wilcoxon rank-sum test. A probe and the containing region were called *differentially methylated* if the p-value of the probe t-statistic was at most 0.05 (uncorrected for multiple testing), log2-fold change between the groups was at least 0.25, and the false discovery rates (FDR) of the region-level statistic was at most 0.2. False positives due to multiple testing are than controlled using the FDR [103] using the method of Benjamini and Hochberg [104].

Figures 1, 2, 3, 4, S1, S2, S3, S4, S5 and S6 were obtained using the UCSC genome browser (http://genome.ucsc.edu/). They depict whole-chromosome views of tracks composed of average normalized probe intensity differences between groups and Pearson’s correlation coefficient (r) between cytokine levels in plasma and normalized MeDIP microarray probe intensities. Similarly to methylation differences, region-level statistical significance was calculated as enrichment probe correlations close to 1 or −1 among the probes in each 1000 bp region partition of the loci tiled with probes using the Wilcoxon rank-sum test. A probe and the containing region were called *correlated* with cytokine levels if the p-value of the correlation was at most 0.05 (uncorrected for multiple testing), the absolute value of the coefficient derived from the linear model used to calculate the p-value was at least 0.5 and the false discovery rates (FDR) of the region-level statistic was at most 0.2. Cytokine levels in plasma were obtained from previous work [32] for the same set of individuals from which DNA methylation profiles were derived.

The identification of regulatory elements contained in the cytokine and TF loci shown in the figures and tables were obtained using the Encyclopedia of DNA Elements (ENCODE) tracks from the UCSC genome browser (http://genome.ucsc.edu/ENCODE/). The transcription factors binding sites and DNase hypersensitive sites (cluster) shown were obtained from ChIP-seq data done on 24 different cell lines. The histone marks shown were also obtained from ChIP-seq data done on 8 different cell lines but we selected only two cell lines relevant to the lymphocytes analyzed in our study, Gm12878 and K562.

The microarray data are available at http://www.ncbi.nlm.nih.gov/geo under the accession number GSE47193.

### Microarray Validation

#### Bisulfite treatment and pyrosequencing

1 µg of EcoRI digested DNA was subjected to bisulfite treatment as previously described [105]. Loci were selected for further analysis from the locations of probes whose normalized intensities were significantly different between the groups (see Microarray Analysis). PCR amplifications were performed in two steps: 25 ng of bisulfite DNA were used for the outside PCR and 1 µl of this DNA for the nested PCR. The outside and nested PCR were performed using HotStarTaq DNA polymerase (Qiagen, Germantown, United States). For pyrosequencing analysis, 25 µl of the nested bisulfite-PCR products were processed according to the manufacturer’s standard protocol (Biotage, Charlotte, United States). Sequencing reactions were performed with a PyroMark Gold Q24 Reagent Kit (Qiagen (Biotage), Germantown, United States) according to the manufacturer’s instructions. The percentage methylation at each CpG site was calculated from the raw data by use of PyroMark Q24-CpG Software (Biotage, Charlotte, United States). The mean methylation of all CG sites analyzed in each region was than tested for correlation with the previously obtained cytokine levels in plasma using Pearson correlation.

## Supporting Information

Figure S1 **DNA methylation differences between CPA (n = 8) and control (n = 12) groups in pro-inflammatory cytokines IL-6 and IL-1α loci in T cells and monocytes.** Expanded views from the UCSC genome browser of IL-6 **(A)** and IL-1α **(B)** loci located on chromosomes 7 and 2 are depicted. The first two tracks shows the average MeDIP probe fold differences (Log_2_) between chronic physical aggressive (CPA) and controls groups and the average Pearson correlation coefficient values calculated between the methylation levels of each probe estimated from the microarray and the plasma IL-6 **(A)** and IL-1α **(B)** levels obtained from the same subject (n = 20) in monocytes. The following tracks show the same set of results but those obtained from T cells. In black are probes that are more methylated and in gray are those that are less methylated in the CPA group. In red are probes whose methylation level correlate positively with the cytokine level in plasma and in green are those that correlated negatively. Highlighted in blue are regions significantly differentially methylated between the groups in T cells, in red in monocytes and in purple in both cell type. The next track (CpG island) shows the location of the CpG islands (CG frequency >0.6) found in the IL-6 **(A)** and IL-1α **(B)** loci. The regulatory element from ENCODE identified in these regions (see methods) are shown in the additional tracks. First, shown with black lines, is the location of DNase hypersensitive clusters where black indicate strong signal and grey a weaker signal from ChIP-seq data in 24 cell lines. Second, is the location of transcription factors (TF) identified from ChIP-seq data in 24 cell lines where black indicate a strong and grey weaker signal occupancy. The last tracks, identified the level of enrichment of three histone marks determined from ChIP-seq assay, histone 3 lysine 4 tri- and mono-methylation as well as histone 3 lysine 27 acetylation in two cell lines, GM12878 (pink) and K562 (blue).
(TIFF)

Figure S2 **DNA methylation differences between CPA (n = 8) and control (n = 12) groups in anti-inflammatory cytokines IL-4 and IL-10 loci in monocytes and T cells.** Expanded views from the UCSC genome browser of IL-4 **(A)** and IL-10 **(B)** loci located on chromosomes 5 and 1 are depicted. The first two tracks shows the average MeDIP probe fold differences (Log_2_) between chronic physical aggressive (CPA) and controls groups and the average Pearson correlation coefficient values calculated between the methylation levels of each probe estimated from the microarray and the plasma IL-4 **(A)** and IL-10 **(B)** levels obtained from the same subject (n = 20) in monocytes. The following tracks show the same set of results but those obtained from T cells. In black are probes that are more methylated and in gray are those that are less methylated in the CPA group. In red are probes whose methylation level correlate positively with the cytokine level in plasma and in green are those that correlated negatively. Highlighted in blue are regions significantly differentially methylated between the groups in T cells, in red in monocytes and in purple in both cell type. The next track (CpG island) shows the location of the CpG islands (CG frequency >0.6) found in the IL-4 **(A)** and IL-10 **(B)** loci. The regulatory element from ENCODE identified in these regions (see methods) are shown in the additional tracks. First, shown with black lines, is the location of DNase hypersensitive clusters where black indicate strong signal and grey a weaker signal from ChIP-seq data in 24 cell lines. Second, is the location of transcription factors (TF) identified from ChIP-seq data in 24 cell lines where black indicate a strong and grey weaker signal occupancy. The last tracks, identified the level of enrichment of three histone marks determined from ChIP-seq assay, histone 3 lysine 4 tri- and mono-methylation as well as histone 3 lysine 27 acetylation in two cell lines, GM12878 (pink) and K562 (blue).
(TIFF)

Figure S3 **DNA methylation differences between CPA (n = 8) and control (n = 12) groups in pro-inflammatory chemokine IL-8 locus in T cells and monocytes.** Expanded view from the UCSC genome browser of IL-8 locus located on chromosomes 4 is depicted. The first two tracks shows the average MeDIP probe fold differences (Log_2_) between chronic physical aggressive (CPA) and controls groups and the average Pearson correlation coefficient values calculated between the methylation levels of each probe estimated from the microarray and the plasma IL-8 levels obtained from the same subject (n = 20) in monocytes. The following tracks show the same set of results but those obtained from T cells. In black are probes that are more methylated and in gray are those that are less methylated in the CPA group. In red are probes whose methylation level correlate positively with the cytokine level in plasma and in green are those that correlated negatively. Highlighted in blue are regions significantly differentially methylated between the groups in T cells, in red in monocytes and in purple in both cell type. The next track (CpG island) shows the location of the CpG islands (CG frequency >0.6) found in the IL-8 loci. The regulatory element from ENCODE identified in these regions (see methods) are shown in the additional tracks. First, shown with black lines, is the location of DNase hypersensitive clusters where black indicate strong signal and grey a weaker signal from ChIP-seq data in 24 cell lines. Second, is the location of transcription factors (TF) identified from ChIP-seq data in 24 cell lines where black indicate a strong and grey weaker signal occupancy. The last tracks, identified the level of enrichment of three histone marks determined from ChIP-seq assay, histone 3 lysine 4 tri- and mono-methylation as well as histone 3 lysine 27 acetylation in two cell lines, GM12878 (pink) and K562 (blue).
(TIFF)

Figure S4 **DNA methylation differences between CPA (n = 8) and control (n = 12) groups in cytokine’s transcription factor NFkB1 in T cells and monocytes.** Expanded view from the UCSC genome browser of NFkB1 locus located on chromosomes 4 is depicted. The first two tracks shows the average MeDIP probe fold differences (Log_2_) between chronic physical aggressive (CPA) and controls groups and the average Pearson correlation coefficient values calculated between the methylation levels of each probe estimated from the microarray and the plasma levels of the cytokines it regulates (IL-1α, IL-6, IL-8 and IL-10) obtained from the same subject (n = 20) in monocytes. The following tracks show the same set of results but those obtained from T cells. In black are probes that are more methylated and in gray are those that are less methylated in the CPA group. In red are probes whose methylation level correlate positively with the cytokine level in plasma and in green are those that correlated negatively. Highlighted in blue are regions significantly differentially methylated between the groups in T cells, in red in monocytes and in purple in both cell type. The next track (CpG island) shows the location of the CpG islands (CG frequency >0.6) found in the NFkB1 loci. The regulatory element from ENCODE identified in these regions (see methods) are shown in the additional tracks. First, shown with black lines, is the location of DNase hypersensitive clusters where black indicate strong signal and grey a weaker signal from ChIP-seq data in 24 cell lines. Second, is the location of transcription factors (TF) identified from ChIP-seq data in 24 cell lines where black indicate a strong and grey weaker signal occupancy. The last tracks, identified the level of enrichment of three histone marks determined from ChIP-seq assay, histone 3 lysine 4 tri- and mono-methylation as well as histone 3 lysine 27 acetylation in two cell lines, GM12878 (pink) and K562 (blue).
(TIFF)

Figure S5 **DNA methylation differences between CPA (n = 8) and control (n = 12) groups in cytokine’s transcription factor NFAT5 in T cells and monocytes.** Expanded view from the UCSC genome browser of NFAT5 locus located on chromosomes 16 is depicted. The first two tracks shows the average MeDIP probe fold differences (Log_2_) between chronic physical aggressive (CPA) and controls groups and the average Pearson correlation coefficient values calculated between the methylation levels of each probe estimated from the microarray and the plasma levels of the cytokines it regulates (IL-8 and IL-4) obtained from the same subject (n = 20) in monocytes. The following tracks show the same set of results but those obtained from T cells. In black are probes that are more methylated and in gray are those that are less methylated in the CPA group. In red are probes whose methylation level correlate positively with the cytokine level in plasma and in green are those that correlated negatively. Highlighted in blue are regions significantly differentially methylated between the groups in T cells, in red in monocytes and in purple in both cell type. The next track (CpG island) shows the location of the CpG islands (CG frequency >0.6) found in the NFAT5 loci. The regulatory element from ENCODE identified in these regions (see methods) are shown in the additional tracks. First, shown with black lines, is the location of DNase hypersensitive clusters where black indicate strong signal and grey a weaker signal from ChIP-seq data in 24 cell lines. Second, is the location of transcription factors (TF) identified from ChIP-seq data in 24 cell lines where black indicate a strong and grey weaker signal occupancy. The last tracks, identified the level of enrichment of three histone marks determined from ChIP-seq assay, histone 3 lysine 4 tri- and mono-methylation as well as histone 3 lysine 27 acetylation in two cell lines, GM12878 (pink) and K562 (blue).
(TIFF)

Figure S6 **DNA methylation differences between CPA (n = 8) and control (n = 12) groups in cytokine’s transcription factor STAT6 in T cells and monocytes.** Expanded view from the UCSC genome browser of STAT6 locus located on chromosomes 12 is depicted. The first two tracks shows the average MeDIP probe fold differences (Log_2_) between chronic physical aggressive (CPA) and controls groups and the average Pearson correlation coefficient values calculated between the methylation levels of each probe estimated from the microarray and the plasma levels of the cytokines it regulates (IL-4 and IL-10) obtained from the same subject (n = 20) in monocytes. The following tracks show the same set of results but those obtained from T cells. In black are probes that are more methylated and in gray are those that are less methylated in the CPA group. In red are probes whose methylation level correlate positively with the cytokine level in plasma and in green are those that correlated negatively. Highlighted in blue are regions significantly differentially methylated between the groups in T cells, in red in monocytes and in purple in both cell type. The next track (CpG island) shows the location of the CpG islands (CG frequency >0.6) found in the STAT6 loci. The regulatory element from ENCODE identified in these regions (see methods) are shown in the additional tracks. First, shown with black lines, is the location of DNase hypersensitive clusters where black indicate strong signal and grey a weaker signal from ChIP-seq data in 24 cell lines. Second, is the location of transcription factors (TF) identified from ChIP-seq data in 24 cell lines where black indicate a strong and grey weaker signal occupancy. The last tracks, identified the level of enrichment of three histone marks determined from ChIP-seq assay, histone 3 lysine 4 tri- and mono-methylation as well as histone 3 lysine 27 acetylation in two cell lines, GM12878 (pink) and K562 (blue).
(TIFF)

Figure S7 **Schematic overview of the recruitment process.**
(TIFF)

Table S1 **List of cell lines and symbols included in the transcription factor binding site data from ENCODE used in the figures generated from UCSC genome browser.**
(DOCX)

Spreadsheet S1 **List of differentially methylated probes predicted from the cytokine and TF microarrays in T cells and monocytes.**
(XLSX)
