# Supplementary figures and images for: Differential DNA Methylation Regions in Cytokine and Transcription Factor Genomic Loci Associate with Childhood Physical Aggression
Source: PLoS One. 2013 Aug 19;8(8):e71691. doi: 10.1371/journal.pone.0071691 (PMC3747262; doi:10.1371/journal.pone.0071691)

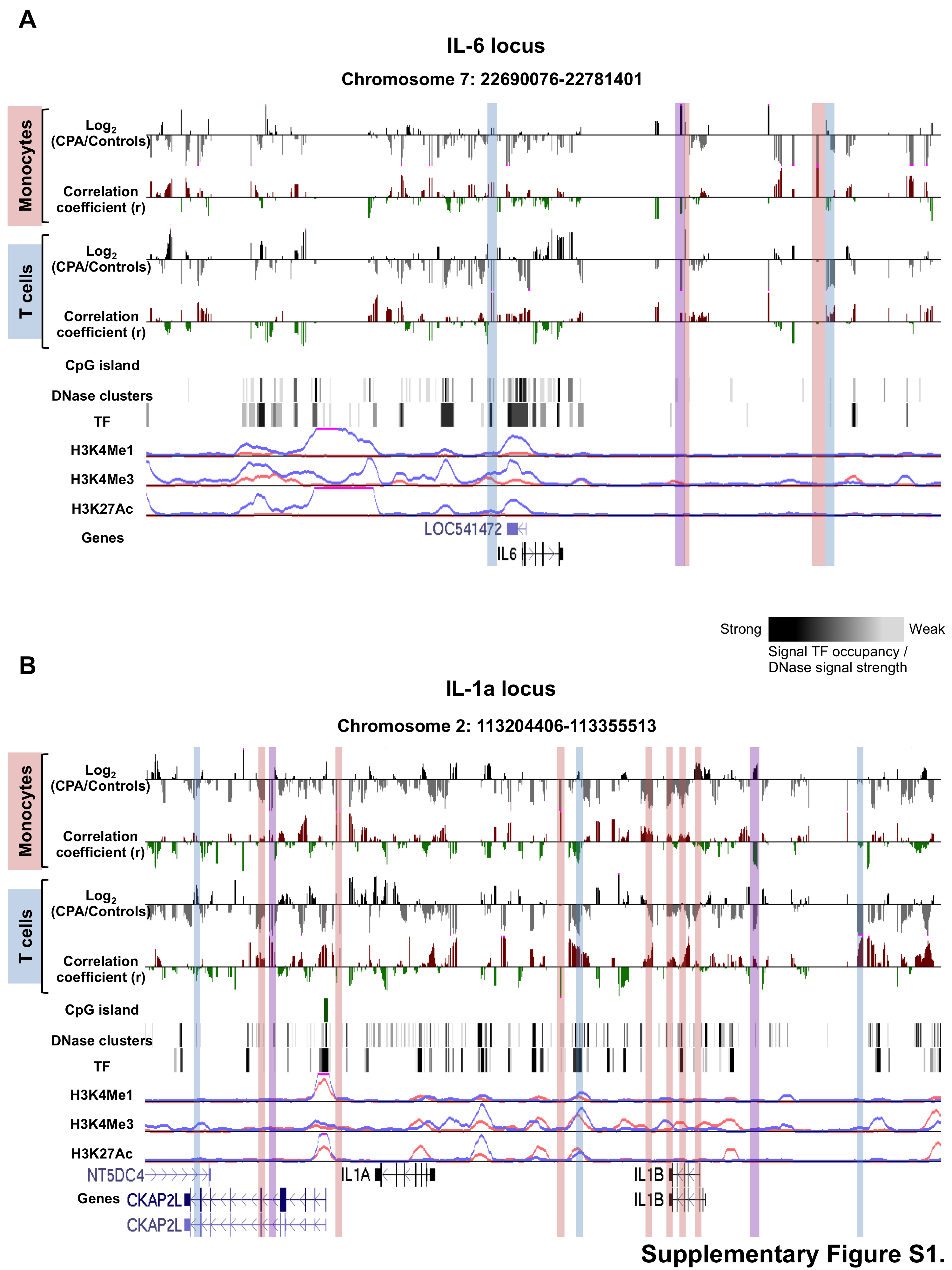

Supplement: Figure S1 — DNA methylation differences between CPA (n = 8) and control (n = 12) groups in pro-inflammatory cytokines IL-6 and IL-1α loci in T cells and monocytes. Expanded views from the UCSC genome browser of IL-6 (A) and IL-1α (B) loci located on chromosomes 7 and 2 are depicted. The first two tracks shows the average MeDIP probe fold differences (Log2) between chronic physical aggressive (CPA) and controls groups and the average Pearson correlation coefficient values calculated between the methylation levels of each probe estimated from the microarray and the plasma IL-6 (A) and IL-1α (B) levels obtained from the same subject (n = 20) in monocytes. The following tracks show the same set of results but those obtained from T cells. In black are probes that are more methylated and in gray are those that are less methylated in the CPA group. In red are probes whose methylation level correlate positively with the cytokine level in plasma and in green are those that correlated negatively. Highlighted in blue are regions significantly differentially methylated between the groups in T cells, in red in monocytes and in purple in both cell type. The next track (CpG island) shows the location of the CpG islands (CG frequency >0.6) found in the IL-6 (A) and IL-1α (B) loci. The regulatory element from ENCODE identified in these regions (see methods) are shown in the additional tracks. First, shown with black lines, is the location of DNase hypersensitive clusters where black indicate strong signal and grey a weaker signal from ChIP-seq data in 24 cell lines. Second, is the location of transcription factors (TF) identified from ChIP-seq data in 24 cell lines where black indicate a strong and grey weaker signal occupancy. The last tracks, identified the level of enrichment of three histone marks determined from ChIP-seq assay, histone 3 lysine 4 tri- and mono-methylation as well as histone 3 lysine 27 acetylation in two cell lines, GM12878 (pink) and K562 (blue). (TIFF) [file pone.0071691.s001.tiff]

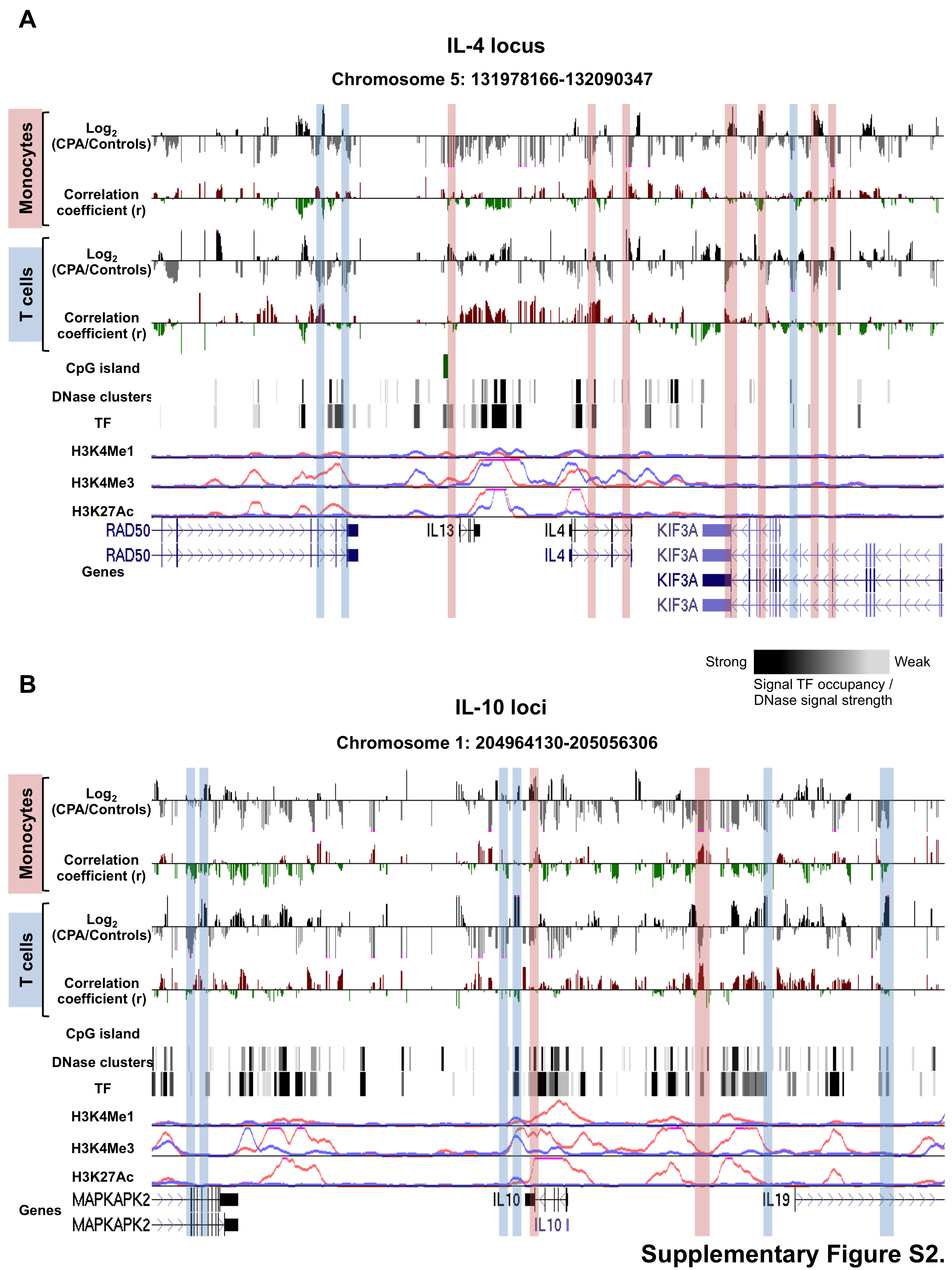

Supplement: Figure S2 — DNA methylation differences between CPA (n = 8) and control (n = 12) groups in anti-inflammatory cytokines IL-4 and IL-10 loci in monocytes and T cells. Expanded views from the UCSC genome browser of IL-4 (A) and IL-10 (B) loci located on chromosomes 5 and 1 are depicted. The first two tracks shows the average MeDIP probe fold differences (Log2) between chronic physical aggressive (CPA) and controls groups and the average Pearson correlation coefficient values calculated between the methylation levels of each probe estimated from the microarray and the plasma IL-4 (A) and IL-10 (B) levels obtained from the same subject (n = 20) in monocytes. The following tracks show the same set of results but those obtained from T cells. In black are probes that are more methylated and in gray are those that are less methylated in the CPA group. In red are probes whose methylation level correlate positively with the cytokine level in plasma and in green are those that correlated negatively. Highlighted in blue are regions significantly differentially methylated between the groups in T cells, in red in monocytes and in purple in both cell type. The next track (CpG island) shows the location of the CpG islands (CG frequency >0.6) found in the IL-4 (A) and IL-10 (B) loci. The regulatory element from ENCODE identified in these regions (see methods) are shown in the additional tracks. First, shown with black lines, is the location of DNase hypersensitive clusters where black indicate strong signal and grey a weaker signal from ChIP-seq data in 24 cell lines. Second, is the location of transcription factors (TF) identified from ChIP-seq data in 24 cell lines where black indicate a strong and grey weaker signal occupancy. The last tracks, identified the level of enrichment of three histone marks determined from ChIP-seq assay, histone 3 lysine 4 tri- and mono-methylation as well as histone 3 lysine 27 acetylation in two cell lines, GM12878 (pink) and K562 (blue). (TIFF) [file pone.0071691.s002.tiff]

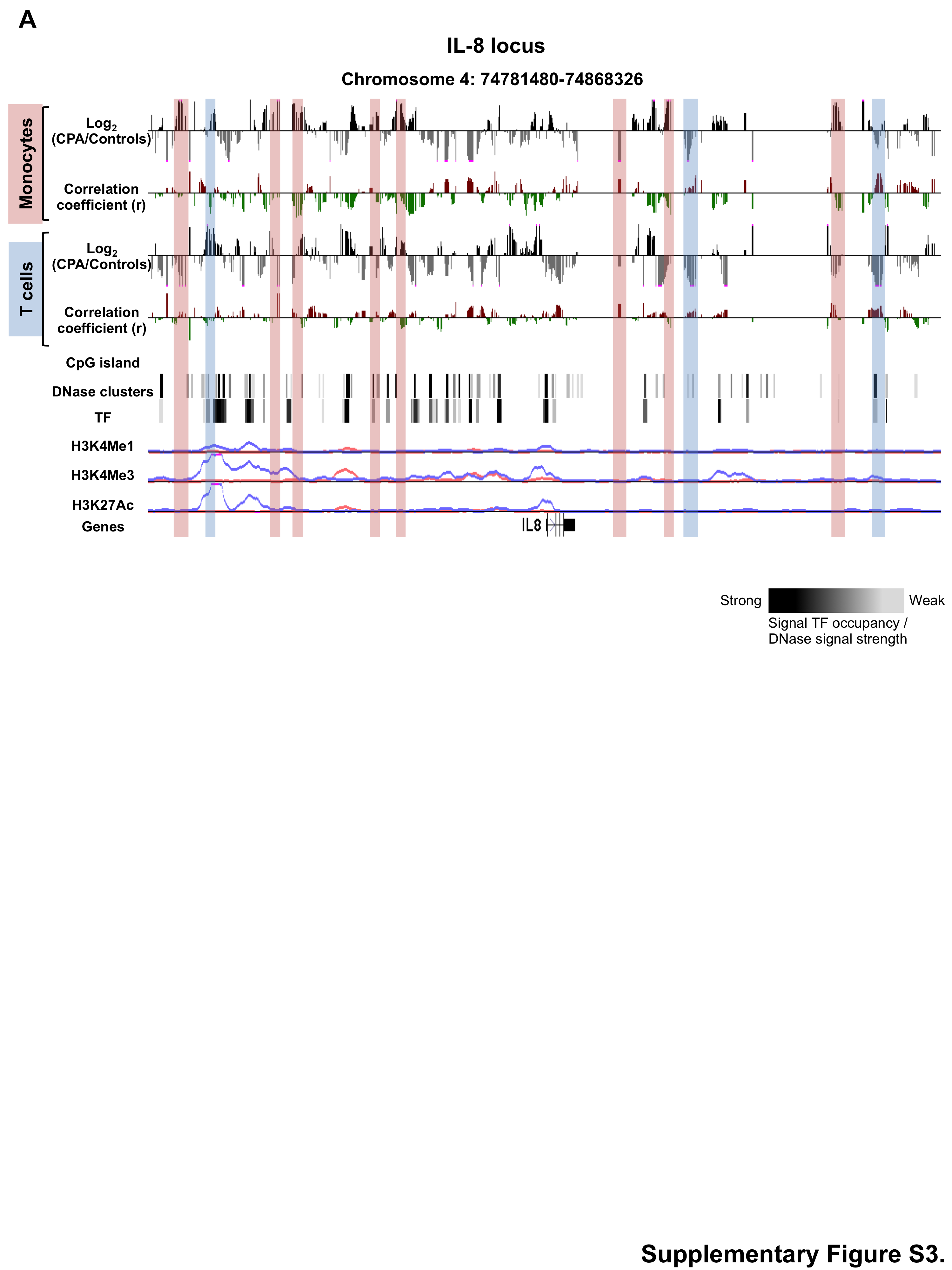

Supplement: Figure S3 — DNA methylation differences between CPA (n = 8) and control (n = 12) groups in pro-inflammatory chemokine IL-8 locus in T cells and monocytes. Expanded view from the UCSC genome browser of IL-8 locus located on chromosomes 4 is depicted. The first two tracks shows the average MeDIP probe fold differences (Log2) between chronic physical aggressive (CPA) and controls groups and the average Pearson correlation coefficient values calculated between the methylation levels of each probe estimated from the microarray and the plasma IL-8 levels obtained from the same subject (n = 20) in monocytes. The following tracks show the same set of results but those obtained from T cells. In black are probes that are more methylated and in gray are those that are less methylated in the CPA group. In red are probes whose methylation level correlate positively with the cytokine level in plasma and in green are those that correlated negatively. Highlighted in blue are regions significantly differentially methylated between the groups in T cells, in red in monocytes and in purple in both cell type. The next track (CpG island) shows the location of the CpG islands (CG frequency >0.6) found in the IL-8 loci. The regulatory element from ENCODE identified in these regions (see methods) are shown in the additional tracks. First, shown with black lines, is the location of DNase hypersensitive clusters where black indicate strong signal and grey a weaker signal from ChIP-seq data in 24 cell lines. Second, is the location of transcription factors (TF) identified from ChIP-seq data in 24 cell lines where black indicate a strong and grey weaker signal occupancy. The last tracks, identified the level of enrichment of three histone marks determined from ChIP-seq assay, histone 3 lysine 4 tri- and mono-methylation as well as histone 3 lysine 27 acetylation in two cell lines, GM12878 (pink) and K562 (blue). (TIFF) [file pone.0071691.s003.tiff]

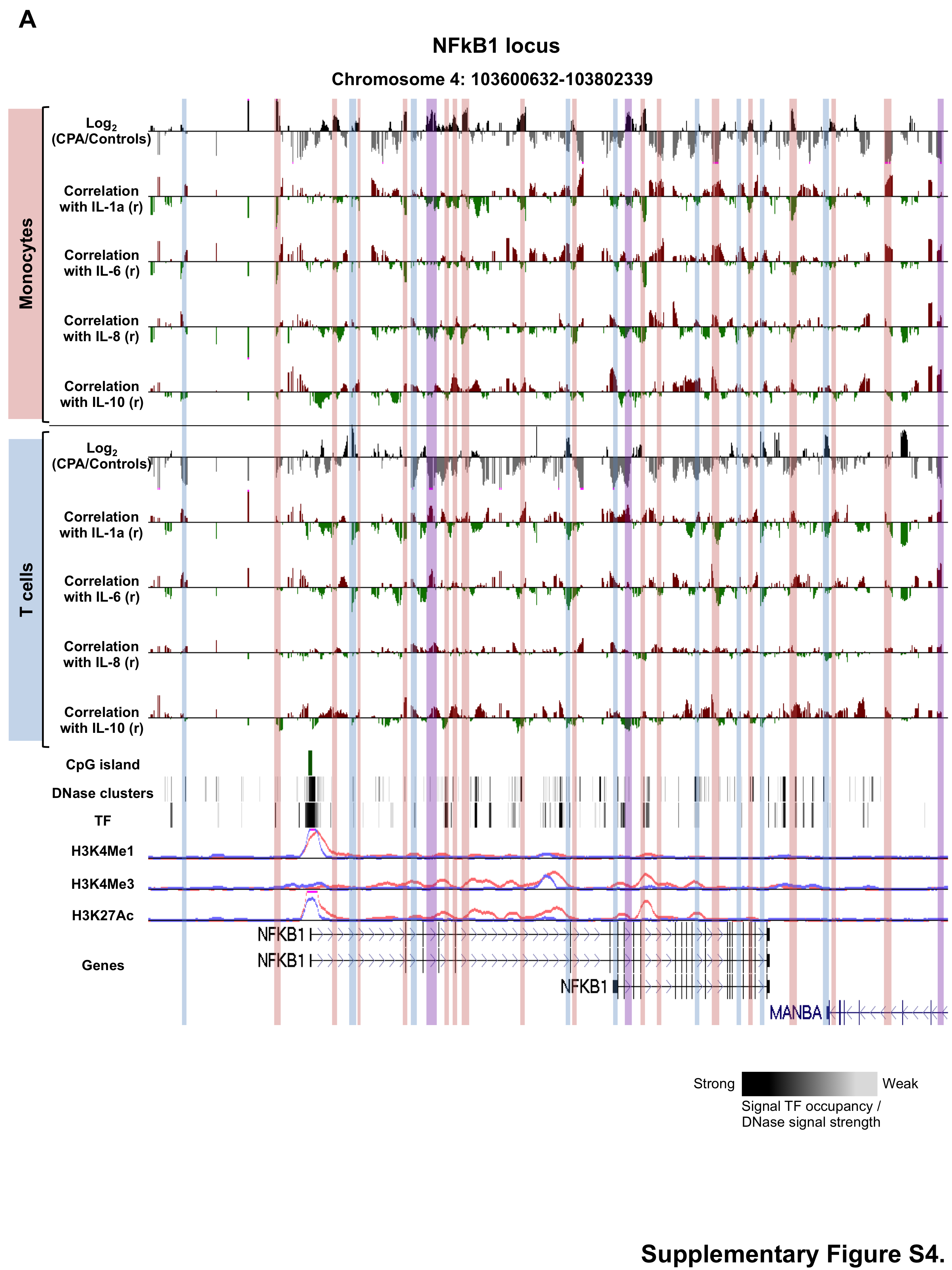

Supplement: Figure S4 — DNA methylation differences between CPA (n = 8) and control (n = 12) groups in cytokine’s transcription factor NFkB1 in T cells and monocytes. Expanded view from the UCSC genome browser of NFkB1 locus located on chromosomes 4 is depicted. The first two tracks shows the average MeDIP probe fold differences (Log2) between chronic physical aggressive (CPA) and controls groups and the average Pearson correlation coefficient values calculated between the methylation levels of each probe estimated from the microarray and the plasma levels of the cytokines it regulates (IL-1α, IL-6, IL-8 and IL-10) obtained from the same subject (n = 20) in monocytes. The following tracks show the same set of results but those obtained from T cells. In black are probes that are more methylated and in gray are those that are less methylated in the CPA group. In red are probes whose methylation level correlate positively with the cytokine level in plasma and in green are those that correlated negatively. Highlighted in blue are regions significantly differentially methylated between the groups in T cells, in red in monocytes and in purple in both cell type. The next track (CpG island) shows the location of the CpG islands (CG frequency >0.6) found in the NFkB1 loci. The regulatory element from ENCODE identified in these regions (see methods) are shown in the additional tracks. First, shown with black lines, is the location of DNase hypersensitive clusters where black indicate strong signal and grey a weaker signal from ChIP-seq data in 24 cell lines. Second, is the location of transcription factors (TF) identified from ChIP-seq data in 24 cell lines where black indicate a strong and grey weaker signal occupancy. The last tracks, identified the level of enrichment of three histone marks determined from ChIP-seq assay, histone 3 lysine 4 tri- and mono-methylation as well as histone 3 lysine 27 acetylation in two cell lines, GM12878 (pink) and K562 (blue). (TIFF) [file pone.0071691.s004.tiff]

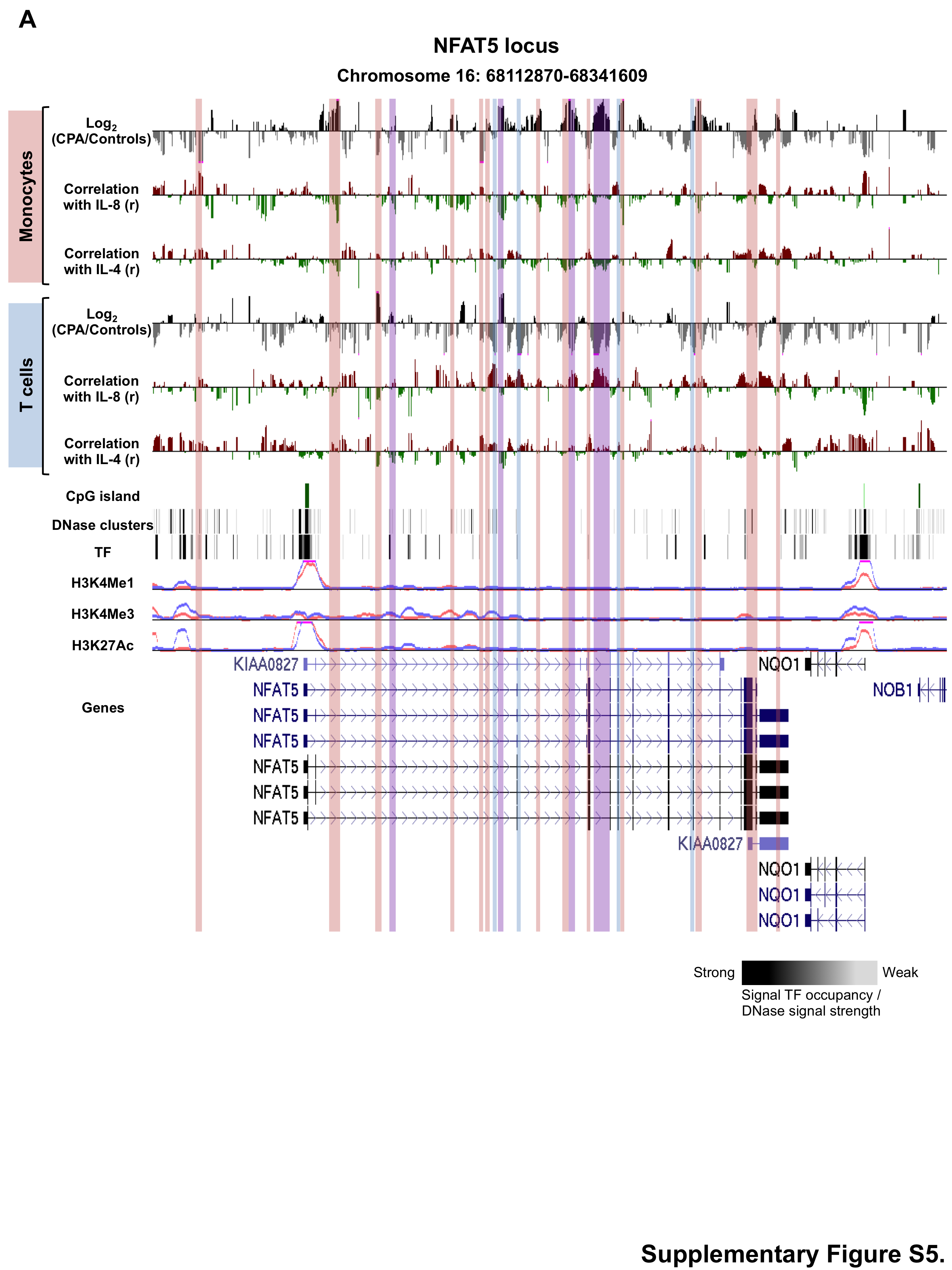

Supplement: Figure S5 — DNA methylation differences between CPA (n = 8) and control (n = 12) groups in cytokine’s transcription factor NFAT5 in T cells and monocytes. Expanded view from the UCSC genome browser of NFAT5 locus located on chromosomes 16 is depicted. The first two tracks shows the average MeDIP probe fold differences (Log2) between chronic physical aggressive (CPA) and controls groups and the average Pearson correlation coefficient values calculated between the methylation levels of each probe estimated from the microarray and the plasma levels of the cytokines it regulates (IL-8 and IL-4) obtained from the same subject (n = 20) in monocytes. The following tracks show the same set of results but those obtained from T cells. In black are probes that are more methylated and in gray are those that are less methylated in the CPA group. In red are probes whose methylation level correlate positively with the cytokine level in plasma and in green are those that correlated negatively. Highlighted in blue are regions significantly differentially methylated between the groups in T cells, in red in monocytes and in purple in both cell type. The next track (CpG island) shows the location of the CpG islands (CG frequency >0.6) found in the NFAT5 loci. The regulatory element from ENCODE identified in these regions (see methods) are shown in the additional tracks. First, shown with black lines, is the location of DNase hypersensitive clusters where black indicate strong signal and grey a weaker signal from ChIP-seq data in 24 cell lines. Second, is the location of transcription factors (TF) identified from ChIP-seq data in 24 cell lines where black indicate a strong and grey weaker signal occupancy. The last tracks, identified the level of enrichment of three histone marks determined from ChIP-seq assay, histone 3 lysine 4 tri- and mono-methylation as well as histone 3 lysine 27 acetylation in two cell lines, GM12878 (pink) and K562 (blue). (TIFF) [file pone.0071691.s005.tiff]

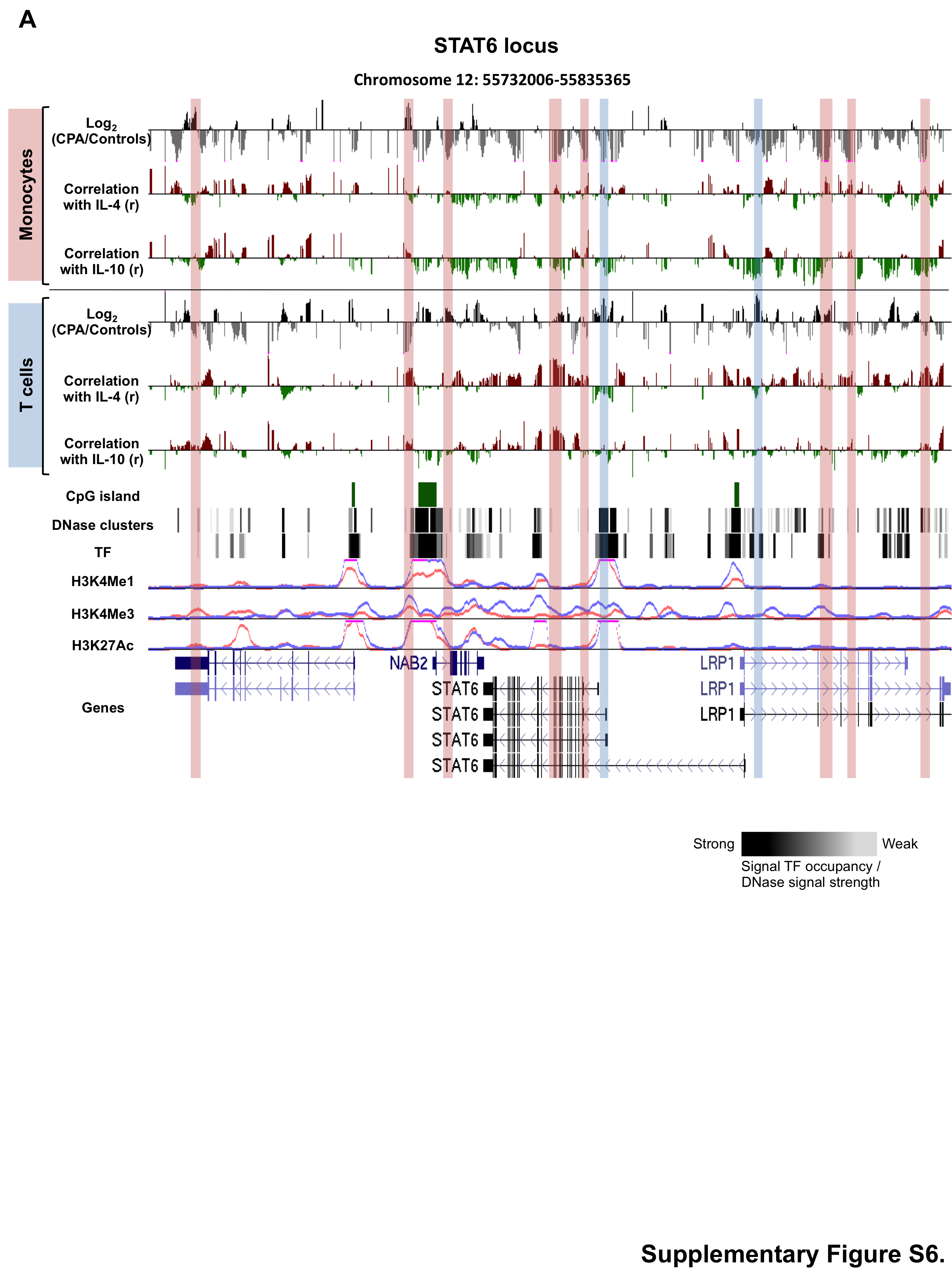

Supplement: Figure S6 — DNA methylation differences between CPA (n = 8) and control (n = 12) groups in cytokine’s transcription factor STAT6 in T cells and monocytes. Expanded view from the UCSC genome browser of STAT6 locus located on chromosomes 12 is depicted. The first two tracks shows the average MeDIP probe fold differences (Log2) between chronic physical aggressive (CPA) and controls groups and the average Pearson correlation coefficient values calculated between the methylation levels of each probe estimated from the microarray and the plasma levels of the cytokines it regulates (IL-4 and IL-10) obtained from the same subject (n = 20) in monocytes. The following tracks show the same set of results but those obtained from T cells. In black are probes that are more methylated and in gray are those that are less methylated in the CPA group. In red are probes whose methylation level correlate positively with the cytokine level in plasma and in green are those that correlated negatively. Highlighted in blue are regions significantly differentially methylated between the groups in T cells, in red in monocytes and in purple in both cell type. The next track (CpG island) shows the location of the CpG islands (CG frequency >0.6) found in the STAT6 loci. The regulatory element from ENCODE identified in these regions (see methods) are shown in the additional tracks. First, shown with black lines, is the location of DNase hypersensitive clusters where black indicate strong signal and grey a weaker signal from ChIP-seq data in 24 cell lines. Second, is the location of transcription factors (TF) identified from ChIP-seq data in 24 cell lines where black indicate a strong and grey weaker signal occupancy. The last tracks, identified the level of enrichment of three histone marks determined from ChIP-seq assay, histone 3 lysine 4 tri- and mono-methylation as well as histone 3 lysine 27 acetylation in two cell lines, GM12878 (pink) and K562 (blue). (TIFF) [file pone.0071691.s006.tiff]

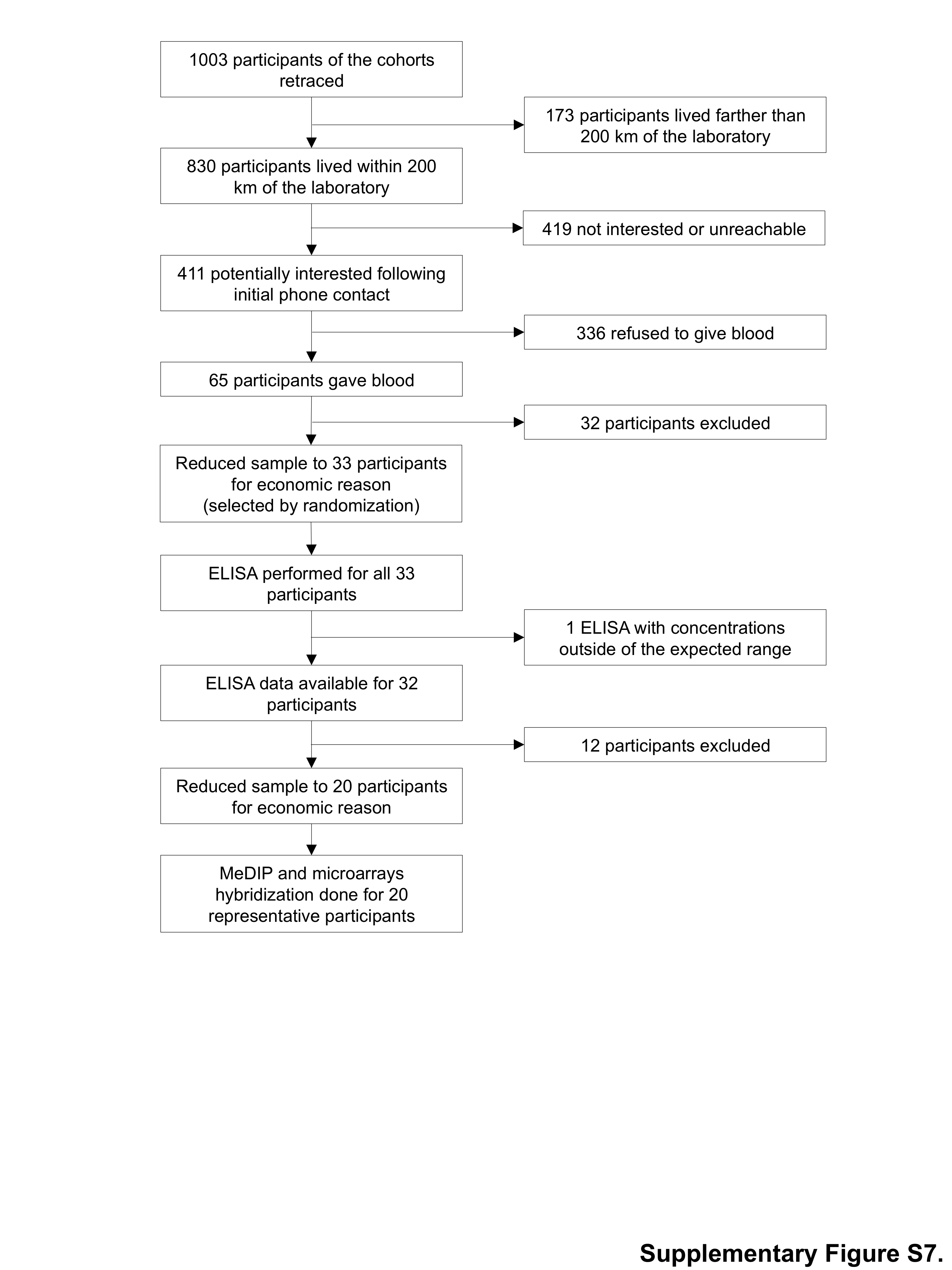

Supplement: Figure S7 — Schematic overview of the recruitment process. (TIFF) [file pone.0071691.s007.tiff]
