# Supplementary material for: Differential DNA Methylation Regions in Cytokine and Transcription Factor Genomic Loci Associate with Childhood Physical Aggression
Source: PLoS One. 2013 Aug 19;8(8):e71691. doi: 10.1371/journal.pone.0071691 (PMC3747262; doi:10.1371/journal.pone.0071691)
Supplement: Table S1 — List of cell lines and symbols included in the transcription factor binding site data from ENCODE used in the figures generated from UCSC genome browser. (DOCX) [file pone.0071691.s008.docx]

**Supplementary Table S1. List of cell lines and symbols included in the transcription factor binding site data from ENCODE used in the figures generated from UCSC genome browser.**

| **Symbol** | **Cell type abbreviation** |
| --- | --- |
| 1 | H1-hESC |
| A | A549 |
| B | BE2_C |
| g | GM10847 |
| G | GM12878 |
| g | GM12891 |
| g | GM12892 |
| g | GM15510 |
| g | GM18505 |
| g | GM18526 |
| g | GM18951 |
| g | GM19099 |
| g | GM19193 |
| h | HEK293(b) |
| H | HeLa-S3 |
| h | HUVEC |
| J | Jurkat |
| K | K562 |
| L | HepG2 |
| N | NB4 |
| p | PANC-1 |
| P | PFSK-1 |
| S | SK-N-MC |
| s | SK-N-SH_RA |
| U | U87 |
